# Functional and spatial proteomics profiling reveals intra- and intercellular signaling crosstalk in colorectal cancer

**DOI:** 10.1016/j.isci.2023.108399

**Published:** 2023-11-04

**Authors:** Christina Plattner, Giorgia Lamberti, Peter Blattmann, Alexander Kirchmair, Dietmar Rieder, Zuzana Loncova, Gregor Sturm, Stefan Scheidl, Marieke Ijsselsteijn, Georgios Fotakis, Asma Noureen, Rebecca Lisandrelli, Nina Böck, Niloofar Nemati, Anne Krogsdam, Sophia Daum, Francesca Finotello, Antonios Somarakis, Alexander Schäfer, Doris Wilflingseder, Miguel Gonzalez Acera, Dietmar Öfner, Lukas A. Huber, Hans Clevers, Christoph Becker, Henner F. Farin, Florian R. Greten, Ruedi Aebersold, Noel F.C.C. de Miranda, Zlatko Trajanoski

**Affiliations:** 1 Biocenter, Institute of Bioinformatics, Medical University of Innsbruck, 6020 Innsbruck, Austria; 2 Department of Biology, Institute of Molecular Systems Biology, ETH Zurich, 8092 Zurich, Switzerland; 3 Department of Visceral, Transplant and Thoracic Surgery, Medical University of Innsbruck, 6020 Innsbruck, Austria; 4 Department of Pathology, Leiden University Medical Center, 2333 ZA Leiden, the Netherlands; 5 Department of Radiology, Leiden University Medical Center, 2333 ZA Leiden, the Netherlands; 6 Institute of Hygiene and Medical Microbiology, Medical University of Innsbruck, 6020 Innsbruck, Austria; 7 Department of Medicine 1, Friedrich-Alexander Universität Erlangen-Nürnberg (FAU) and Universitätsklinikum Erlangen, 91054 Erlangen, Germany; 8 Biocenter, Institute of Cell Biology, Medical University of Innsbruck, 6020 Innsbruck, Austria; 9 Hubrecht Institute, 3584 CT Utrecht, the Netherlands; 10 Institute for Tumor Biology and Experimental Therapy, Georg-Speyer-Haus, 60596 Frankfurt am Main, Germany; 11 Frankfurt Cancer Institute, Goethe University, 60596 Frankfurt am Main, Germany; 12 German Cancer Consortium (DKTK), partner site Frankfurt/Mainz, a partnership with DKFZ Heidelberg, Frankfurt/Mainz, Germany

**Keywords:** Cancer systems biology, Cancer, Proteomics

## Abstract

Precision oncology approaches for patients with colorectal cancer (CRC) continue to lag behind other solid cancers. Functional precision oncology—a strategy that is based on perturbing primary tumor cells from cancer patients—could provide a road forward to personalize treatment. We extend this paradigm to measuring proteome activity landscapes by acquiring quantitative phosphoproteomic data from patient-derived organoids (PDOs). We show that kinase inhibitors induce inhibitor- and patient-specific off-target effects and pathway crosstalk. Reconstruction of the kinase networks revealed that the signaling rewiring is modestly affected by mutations. We show non-genetic heterogeneity of the PDOs and upregulation of stemness and differentiation genes by kinase inhibitors. Using imaging mass-cytometry-based profiling of the primary tumors, we characterize the tumor microenvironment (TME) and determine spatial heterocellular crosstalk and tumor-immune cell interactions. Collectively, we provide a framework for inferring tumor cell intrinsic signaling and external signaling from the TME to inform precision (immuno-) oncology in CRC.

## Highlights

• Functional phosphoproteomics profiling of organoids reveals off-target effects
• Mutations have minor effect on kinase networks rewiring
• Single-cell analysis shows phenotypic and differentiation heterogeneity of organoids
• Spatial single-cell proteomics profiling reveals heterocellular crosstalk

## Graphical abstract

**Figure:**
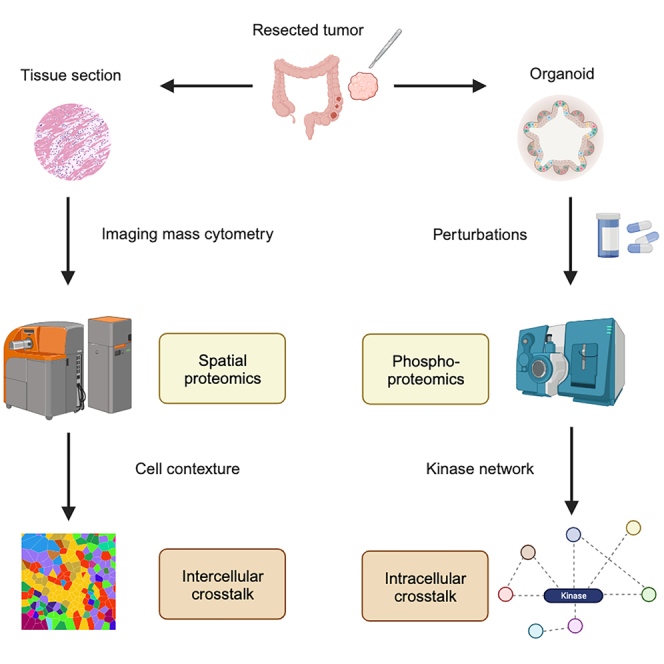

## Introduction

In the past two decades, tremendous advances have been made in both cancer biology by identifying recurrent mutations in oncogenic signaling pathways using sequencing technologies and therapeutics by developing targeted drugs specific for these mutations. In colorectal cancer (CRC), one of the major cancers with high incidence and where mortality rates are still high,^1^ progress in targeted therapy has been limited, relative to other solid cancers like lung cancer or melanoma.^2^ The genetic heterogeneity^3^ as well as paucity of druggable targets (nearly 50% of all CRCs are driven by undruggable oncogenes of the RAS family with the exception of 3% who harbor *KRAS* G12C mutation)^2^ poses considerable challenges for developing precision oncology approaches for patients with CRC. Moreover, CRC seems to be refractory to therapy with immune checkpoint blockers (ICBs) with the notable exception of CRC tumors characterized by mismatch-repair deficiency or POLE proofreading mutations.^4^ This is somehow paradoxical since CRCs, irrespectively of mismatch-repair status, are known to be under immunological control, as we have shown in the past.^5^

In precision oncology the paradigm is emerging that genomic profiling of the tumor assessed at early intervention (biopsy or resection) does not provide sufficient information to guide therapy. A diagnostic approach that is functional, i.e., measuring responses to perturbations with living cells derived from the specific tumor is expected to provide immediately translatable, personalized treatment information.^6^ Such functional precision medicine approach requires patient-derived models representing the tumors from affected individuals like patient-derived xenografts (PDXs) or patient-derived organoids (PDOs).^7^ However, despite earlier encouraging reports,^8^^,^^9^ conflicting results have been reported regarding the capability of PDOs to predict tumor responses to specific drugs.^10^ Moreover, a recent study showed that despite sensitivity of the PDOs to selected kinase inhibitors, CRC patients from whom these cells originated did not demonstrate objective clinical response to the treatment.^11^ A number of reasons can be attributed to the limited value of PDOs as a tool for functional precision oncology. These include PDO culture success rate, ineligibility of the patients, or limited set of drugs tested.^11^ While improvements for more streamlined experimental design are conceivable and could accelerate the implementation of PDOs to guide treatment decisions, major limitations of this drug-screening approach remain. The readouts of the assays for testing *in vitro* response are based on growth rates of the cells and do not measure changes in the levels of functional status of the corresponding proteins and hence do not provide insights into the mechanisms underlying sensitivity or resistance to a specific drug. Moreover, given the large number of available approved drugs, testing existing drugs for novel therapeutic strategies (drug repurposing) or testing novel combinations even for a limited number of single agents becomes impractical. Thus, an improved approach is needed that identifies key cancer cell vulnerabilities and provides rationale to select drugs/drug combinations. Given the fact that dysfunctional signaling in tumors arises from rewiring of signaling pathways and that nearly all molecularly targeted therapeutics are directed against signaling molecules,^12^ a strategy that focuses on cancer cell signaling measurements in PDOs could offer an alternate road forward.

Such a functional precision oncology strategy based on comprehensive dissection of tumor cell signaling depends on obtaining quantitatively accurate and consistent phosphoproteomics profiles. Recently, data-independent acquisition (DIA) methods emerged as a technology that combines deep proteome coverage with quantitative consistency and accuracy. Specifically, a variant of DIA methods called sequential window acquisition of all theoretical mass spectra (SWATH-MS)^13^ was developed in which all ionized peptides within a specified mass range are fragmented for each sample in a systematic manner^14^ and thereby enable reproducible high-throughput identification and quantification of proteomes across many samples. We reasoned that functional precision profiling using PDOs and SWATH-MS-based quantitative phosphoproteomics would enable patient-level reconstruction of kinase signaling networks and shed light on the intrinsic biology of the CRC cells.

**Figure 1:**
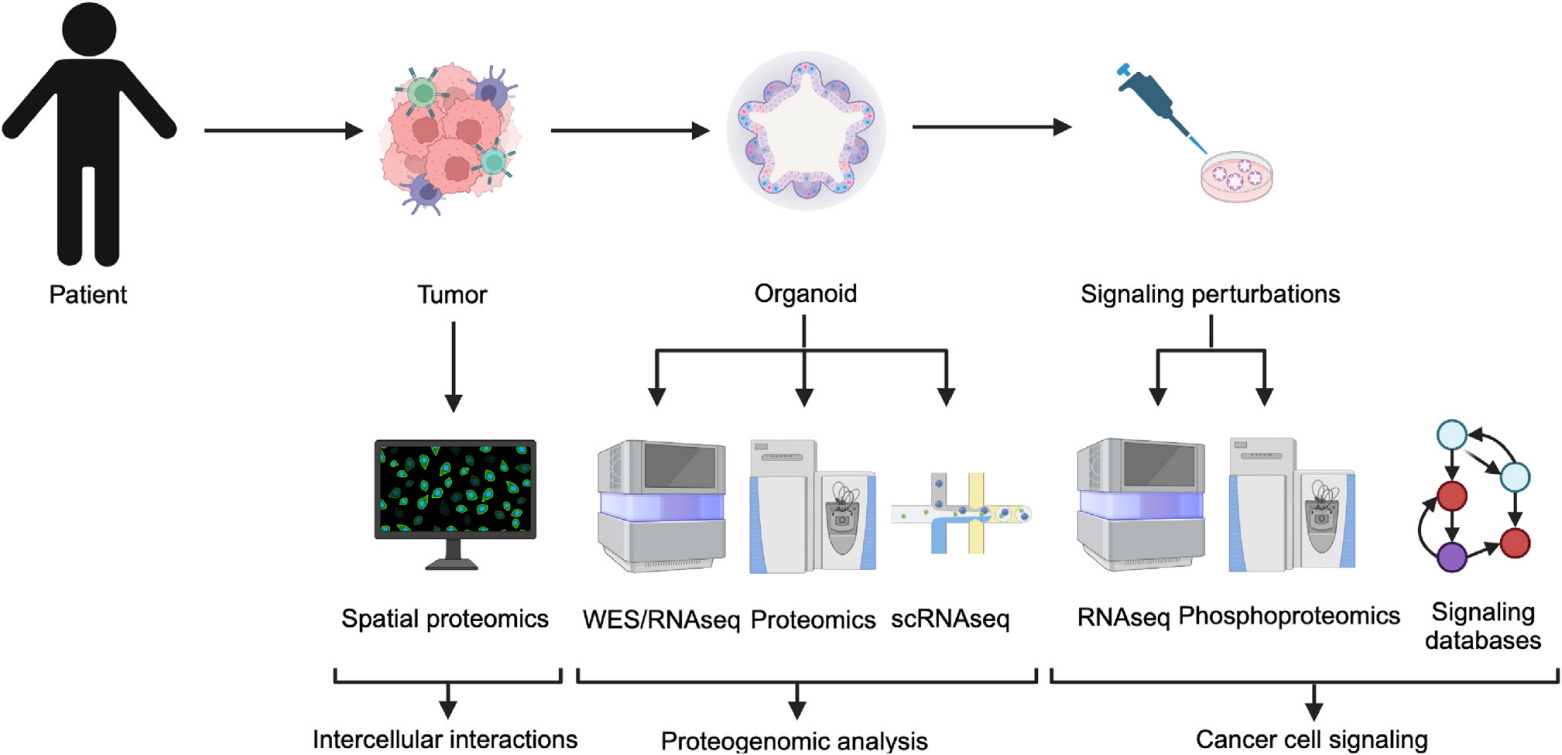
Schematic outline of the overall concept used in this study Multi-modal profiling and multi-omic profiling of tumor specimens and PDOs in a cohort of CRC patients. See also Table S1.

We therefore first established a living biobank of PDOs from CRC patients and carried out steady-state proteogenomic characterization using DNA and RNA sequencing (RNA-seq), and SWATH-MS-based proteomics (Figure 1). We then developed a functional precision oncology approach based on perturbation experiments of the PDOs with kinase inhibitors, quantitative phosphoproteomic measurements, and integration of *a priori* knowledge. We show that kinase inhibitors induce profound off-target effects that impact oncogenic and immune-related pathways. Reconstruction of the topologies of kinase signaling networks showed that patient-specific rewiring is modestly affected by mutations. Moreover, we show non-genetic heterogeneity of the PDOs and upregulation of stemness and differentiation genes by kinase inhibitors. Finally, we complemented our functional precision profiling by IMC-based analysis of the primary tumors that enabled us to quantify spatial heterocellular crosstalk and tumor-immune cell interactions.

## Results

### Proteogenomic characterization of a living biobank

**Figure 2:**
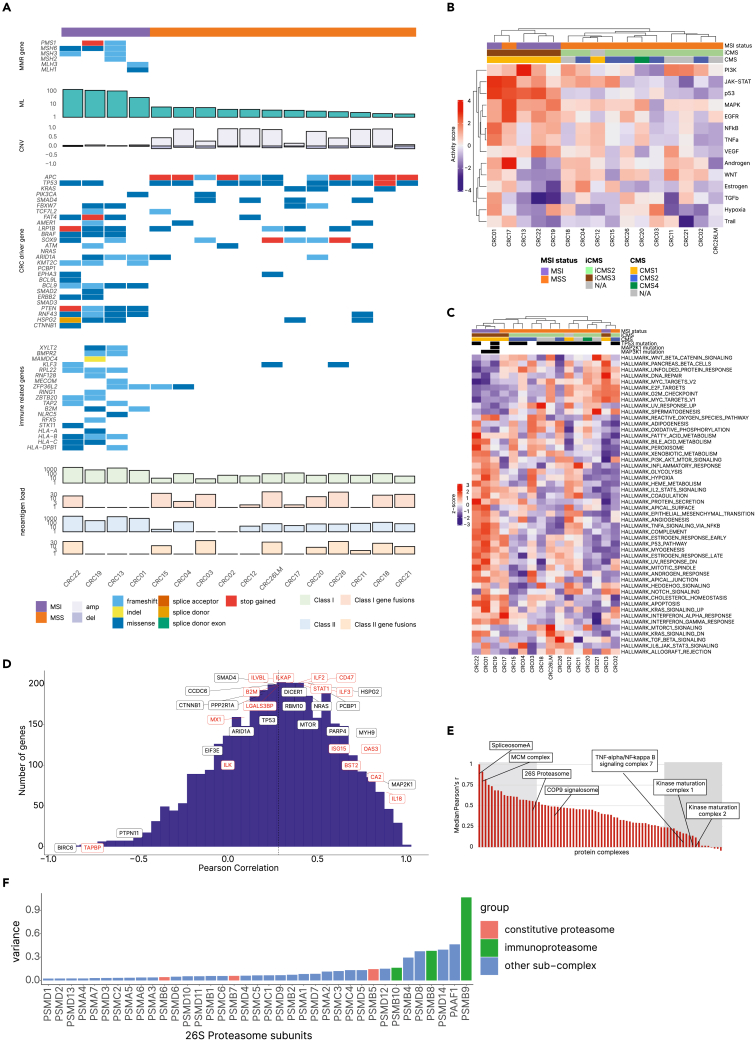
Proteogenomic analysis of PDOs from CRC patients (A) Genetic profiles of the PDOs ordered according to the mutational load (ML). MMR: mismatch-repair. ML: mutational load. CNV: copy number variation. (B) Analysis of the cancer pathways of the PDOs using bulk RNA-seq data and PROGENy. The pathway activity scores are z-scaled and clustered hierarchically by euclidean distance and complete linkage. (C) Pathway analysis of the hallmark gene sets from MSigDB of the PDOs using proteomic data (SWATH-MS). The heatmap shows z scores of enrichment scores derived from Gene Set Variation Analysis (GSVA) and clustered hierarchically by Pearson correlation as distance metric and complete linkage. (D) Correlation analysis between RNA-seq data and proteomics data. The histogram shows gene-wise Pearson correlation between transcriptome and proteome levels. Denoted are driver genes (black) and immune-related genes (red). The average gene-wise Pearson correlation is 0.29 (dashed line). (E) Protein complexes ranked according to the co-abundance observed for complex members. Shaded areas, left: stable complexes (top 25%), right: variable complexes (bottom 25%). MCM: mini chromosome maintenance. COP9: constitutive photomorphogenesis 9. (F) Variance of the protein levels of the 26S proteasome subunits across all PDOs.

We generated and molecularly characterized a living biobank using organoid technology^15^ as the basis for the subsequent analyses. Tumor samples from 15 CRC patients (Table S1), including microsatellite instable (MSI) and microsatellite stable (MSS) tumors were used to generate PDOs which were then characterized using exome sequencing, transcriptome sequencing, and SWATH-MS-based proteomics. Genomic characterization including CRC driver genes^16^ and a panel of immune-related genes frequently mutated in CRC reaffirmed previously reported somatic alterations^17^ and showed that the biobank is representative of CRC (Figure 2A; Figure S1A). As expected, MSS PDOs have few targetable mutations whereas mutations in immune-related genes including mutations in human leukocyte antigen (HLA) class I and class II genes were almost entirely detectable in MSI PDOs (Figure 2A; Table S2). WNT signaling (*CTNNB1* (β-catenin)) and transforming growth factor β (TGF-β) signaling genes (*SMAD2*, *SMAD4*) were mutated in both subtypes. The concordance of variants between primary tumor tissue and the corresponding organoids was in line with previously published datasets (Figure S1B).^18^ The genetic representativeness of PDOs has been previously reported,^19^^,^^20^ but whether organoids can also reflect non-genetic heterogeneity has not been established so far. Therefore, we performed a side-by-side comparison of cell type marker expression in tumor samples and the corresponding PDOs (n = 30) using bulk RNA-seq data from our recent study^18^ (Figures S1C and S1D). The results indicate representativeness of the PDOs (with respect to the epithelial subsets) with their respective tumors of origin.

We then predicted the presence of potential neoantigens including antigens derived from missense mutations and from fusion genes RNA-seq data and our recently developed tool for predicting neoantigens,^21^ using whole exome sequencing (WES) data from organoids and matching peripheral blood mononuclear cells (PBMCs) from the patients and RNA-seq data from the organoids (Figure 2A). Both HLA class I and class II-associated neoantigens from gene fusions were mostly detectable in MSS PDOs. Analyses of the neoantigen landscape showed that neoantigens from tandem duplications from acid ceramidase (ASAH1) were predicted in 25% of the PDOs (Table S2), suggesting that this public neoantigen can be used for developing therapeutic vaccination using off-the-shelf vaccine for this CRC cohort.

Analysis of the steady-state transcriptomic data revealed expression of genes involved in chemokine-mediated signaling pathways, indicating possible crosstalk with immune cells (Figure S2A). We then assembled a panel of genes associated with cancer immunity and immune evasion mechanisms including checkpoint molecules, antigen processing and presentation genes, specific chemokines and cytokines, and tumor cell-specific interferon γ (IFNγ)-related genes.^22^ The expression of these genes was highly heterogeneous in the MSI and MSS samples with HLA class I genes showing increased expression in MSI relatively to MSS PDOs, albeit not statistically significant (Figure S2B). Analysis of cancer pathways using transcriptomic data showed heterogeneous pathway activity and partitioning of the profiles in two main subgroups, one of which was predominantly MSI subtype related with one exception, CRC17 (Figure 2B). This is in concordance with a previous report showing that a small fraction of MSS tumors is transcriptionally more similar to the MSI-H tumors than to the MSS group.^23^ We then performed consensus molecular subtype classification for the traditional (CMS1-CMS4) and for the intrinsic (iCMS2/iCMS3) subtypes and classified CRC17 to the CMS1 and iCMS3 subtype, respectively (Table S1).

Analysis of the protein expression data following SWATH-MS of the PDOs showed 3,723 unique proteins across the samples (Figure S2C). Principal-component analysis and pathway analysis using protein expression data showed partitioning related to the MSI and MSS subtypes (Figure 2C; Figure S2D) with notable exception of CRC13 from the MSI group and CRC17 from the MSS group. Within the MSI group there was a tendency for coordinated upregulation of the immune-related pathways, IFNα response, IFNγ response, and IL2/STAT5 (Figure 2C). Gene-wise correlation analysis between RNA and protein expression (average gene-wise Pearson correlation 0.29, Figure 2D) showed that RNA expression is a poor predictor of protein expression for CRC driver genes and for immune-related genes.

As proteins generally exert their function in coordination with other proteins and often form complexes with correlated subunit abundances,^24^^,^^25^ we analyzed the co-abundance for complex members of 78 complexes with at least 5 protein members across all PDOs (Table S3). Complexes with variable subunit composition included TNFα/NF-κB signaling complex 7 and kinase maturation complexes 1 and 2 (Figure 2E). In our analysis, complexes with invariant subunit composition included spliceosome-A, mini chromosome maintenance (MCM) complex, and 26S proteasome. However, within the 26S proteasome, large variability was observed for the protein complex subunits PSMB9 and PSMB8 of the immunoproteasome (Figure 2F), the expression of which is associated with immune response to ICBs in melanoma.^26^ Noteworthily, the protein level of PSMB9 was decreased in a large fraction of the organoids (Figure S2E).

In summary, steady-state multi-omics profiling of the PDOs revealed molecular heterogeneity within the clinical subgroups of MSI and MSS tumors and showed a number of altered signaling pathways that could determine cellular responses to drug treatment. However, based solely on these multi-omic data, the identification of the mechanisms that shape key tumor vulnerabilities and determine response to targeted therapy remained elusive.

### Functional precision profiling of PDOs reveals off-target effects and pathway crosstalk

**Figure 3:**
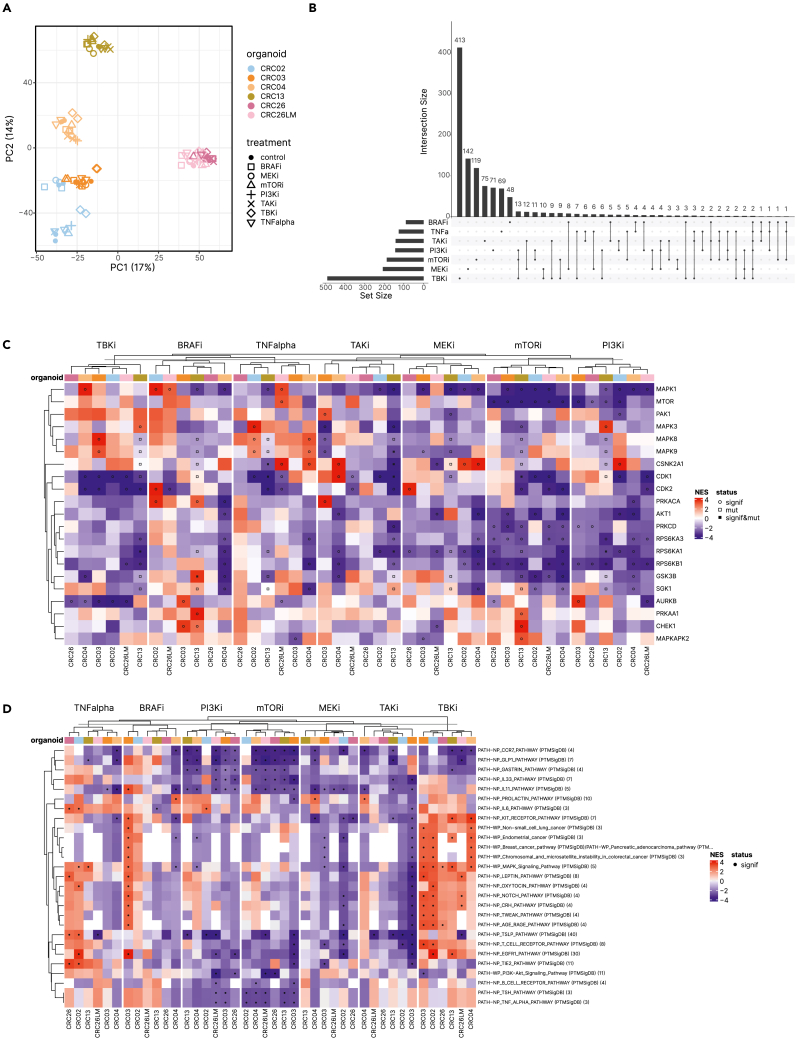
Functional profiling experiments of the PDOs with targeted drugs (A) PCA of the phosphoproteomic data. (B) UpSet plot of regulated phosphopeptides (|log2FC|>1, FDR<0.05) following treatment with specific kinase inhibitors. (C) Heatmap of normalized enrichment scores (NESs) of phosphorylation signatures from PTMSigDB^27^ and SIGNOR^28^ with at least five phosphorylation sites representing changes in kinase activities following treatment of PDOs with specific kinase inhibitors or TNFα (FDR<0.05), clustered by complete linkage of Euclidean distances. Significant changes and mutations are highlighted with circles and squares, respectively. (D) Normalized enrichment scores (NESs) of phosphorylation signatures representing changes in pathway activities following treatment of PDOs with specific kinase inhibitors or TNFα (FDR<0.05, database and number of phosphorylation sites shown in brackets). Significant changes are highlighted with black circles.

In order to investigate the effects of the targeted drugs on specific pro-tumorigenic pathways as well as to identify potential signaling crosstalk with antitumorigenic pathways, we used perturbation experiments and quantitative phosphoproteomic profiling. We carried out perturbation experiments on six PDOs using a panel of kinase inhibitors (BRAFi, MEKi, mTORi, PI3Ki, TAKi, TBKi, Figures S3A and S3B) and one stimulus (TNFα) followed by RNA-seq and SWATH-MS phosphoproteomics measurements. SWATH-MS phosphoproteomics data of the signaling perturbation experiments were highly reproducible (coefficient of variation <10%) and comprised 10,664 phosphopeptides that were mapped to 7,778 unique phosphosites (Figures 3A and S3C). Unsupervised clustering of perturbation-induced changes in the identified phosphosites showed that the responses were mostly specific for PDOs (Figure S3C), suggesting patient-specific rather than treatment-specific phosphoproteomes. The overlap of the regulated phosphopeptides between the treatments was in the lower percentile range (Figure 3B) even for treatments with inhibitors targeting kinases in the same pathway (e.g., the inhibition of BRAF and MEK in the mitogen-activated protein kinase [MAPK] pathway).

We then analyzed 103 kinases using 508 phosphosites that matched phosphosites in a curated database of phosphosite-specific pathway signatures (PTMsigDB)^27^ (Table S3). The analysis revealed highly diverse responses to the perturbations in the PDOs (Figure 3C; Figure S3D). We then analyzed the common treatment effects shared across organoids (Figure S3E). This indicated the expected downregulation of mTOR upon mTORi, of AKT upon PI3Ki, and of “MAPK Signaling Pathway” upon MEKi treatment, and an upregulation of the phosphorylation signatures of TORIN1 upon mTORi treatment and of LY294002 following PI3Ki treatment. However, there were extensive off-target effects of the inhibitors including activation of kinases in non-targeted cascades that were specific for both PDOs and kinase inhibitors. For example, we observed an activation of CDK1 following TAKi treatment in CRC04 and increased CSNK2A1 activity upon MEK inhibition in CRC02 and CRC04 (Figure 3C). Notably, upregulation of CDK1^29^ or CSNK2A1^30^ is associated with worse prognosis in multiple cancer types as well as with suppression of anti-tumor immunity and can thus be considered an unintended adverse off-target effect. Set enrichment analyses with the phosphosite-specific signatures (PTMsigDB)^27^ revealed crosstalk with a number of immune-related pathways, like IL-11, IL-6, and IL-33 pathway (Figure 3D). The pathway crosstalk was PDO- and inhibitor-specific and included increased and reduced pathway activity.

### Mutations are only partially responsible for kinase networks rewiring

**Figure 4:**
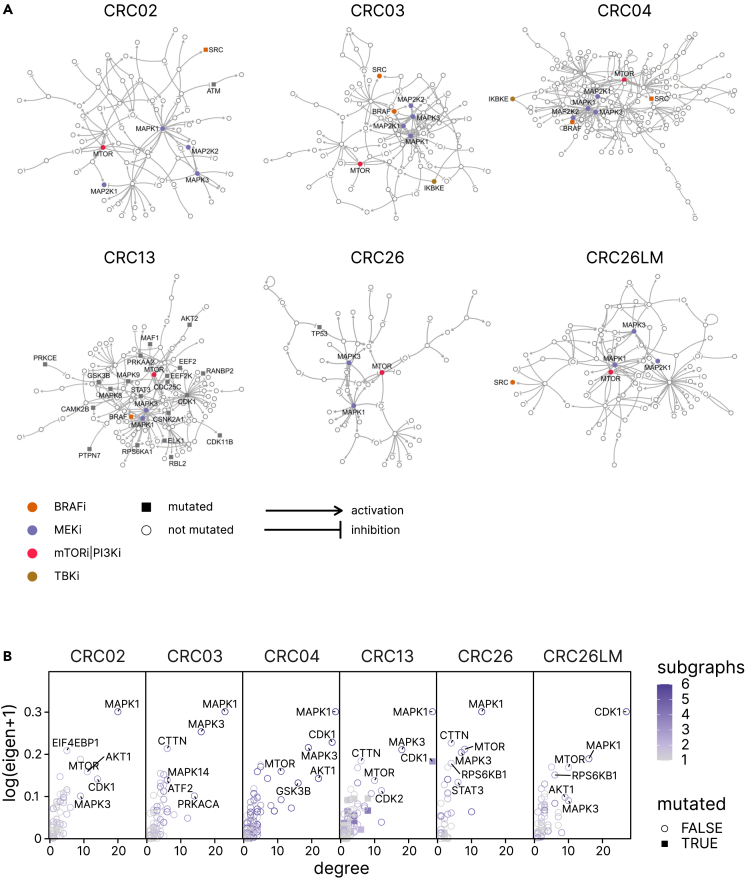
Comparative analysis of the kinase network topologies for the perturbed PDOs (A) Visual representation of the reconstructed kinase networks. Highlighted in color are kinases directly targeted by inhibitors. (B) Eigenvector and degree centrality measures of kinase nodes in the networks shown in (A). Color indicates the number of subgraphs that share a particular node.

The observed off-target activation of kinases in non-targeted cascades and the resulting pathway crosstalk necessitates detailed characterization of the signal transduction network in order to identify optimal targets for effective modulation of the respective pathways. Signal transduction networks are highly adaptable and dynamic, the properties of which are primarily determined by the network topology.^31^ In an attempt to reconstruct signaling networks in individual patients, we developed a computational method using the phosphoproteomic data and *a priori* knowledge of protein-protein interactions (see STAR Methods). Briefly, we assigned kinase activities to nodes and phosphosites to edges of the SIGNOR 2.0 signaling network.^28^ To identify subnetworks probed by the perturbations with kinase inhibitors, only nodes with differential kinase activity based on the enrichment score calculated using PTMSEA^27^ and edges differentially phosphorylated were considered, and the largest module was extracted (see STAR Methods). The individual subnetworks resulting from each perturbation were then amalgamated into a combined kinase signaling network for the corresponding PDO. The kinase signaling networks showed a large extent of heterogeneity with varying numbers of nodes and edges as well as kinase activities and target site phosphorylations (Figure 4A; Figure S4). Strikingly, we found no significant association of mutations with PDO networks (CRC02 pval = 0.18, CRC03 pval = 0.63, CRC04 pval = 1.00, CRC13 pval = 0.61, CRC26 pval = 0.26, CRC26LM pval = 1.00, Fisher’s exact test), suggesting that mutations are only partially responsible for the kinase networks rewiring. In the 5 MSS PDOs harboring between 116 and 230 mutations (coding variants), there were zero (CRC03, CRC26LM), one (CRC04, CRC26), and two nodes (CRC02) with mutated proteins. Even in the MSI PDO (CRC13) with a large number of mutations (2,850 coding variants) there was no significant association of the mutations with the edges in the signaling kinase network (pval = 0.61).

It has been previously shown that graph-based centrality metrics are correlated with the importance of nodes in maintaining network integrity.^32^ We therefore performed comparative analysis of the kinase signaling networks for the PDOs using degree and eigenvector centrality (i.e., the number of edges and the connectivity to highly connected nodes, respectively) values (Figure 4B). As expected, high centrality values were observed for targeted kinases (e.g., mTOR) and for the kinases in the EGFR-RAS-MAPK pathway (e.g., MAPK1), which were present in all of the PDO networks. However, additional nodes like PRKACA or PTPN7 had high centrality values only in some PDOs, further supporting the notion of extensive off-target effects and pathway crosstalk.

Overall, using quantitative phosphoproteomic data from PDOs perturbed with kinase inhibitors, we were able to reconstruct kinase signaling networks at the patient level. Albeit heterogeneous between PDOs, the kinase signaling network topologies revealed intrinsic rewiring that was modestly affected by harboring mutations.

### Single-cell analysis shows phenotypic and differentiation heterogeneity of PDOs

Non-genetic mechanisms like phenotypic plasticity and differentiation status of the tumor cells might have a large impact on adapting the signaling circuitry and leading to disease phenotypes. According to the cancer stem cell hypothesis, tumors are organized in cell hierarchies similar to normal tissues, with stem cells at the apex, giving rise to transient amplifying (TA) progenitor cells that undergo differentiation into several cell lineages.^33^ The revised cancer stem cells model postulates that cancer cells can dynamically shift between a differentiated state and a stem-like state^34^ which in CRC is tightly linked to changes in WNT signaling. Noteworthily, disrupted differentiation is integral to colon carcinogenesis and is a regulator of cellular plasticity. Most CRCs are diagnosed as moderately differentiated^35^ with some cell types being implicated in therapy response. For example, it has been shown that enteroendocrine progenitors support *BRAF*-mutant CRC.^36^ Hence, it appears that the hierarchically organized tumor cell heterogeneity and cell plasticity play key roles in both CRC progression and therapy response as shown recently.^37^ We therefore aimed to determine the hierarchically organized tumor cell heterogeneity in our PDOs and employed single-cell RNA sequencing.

**Figure 5:**
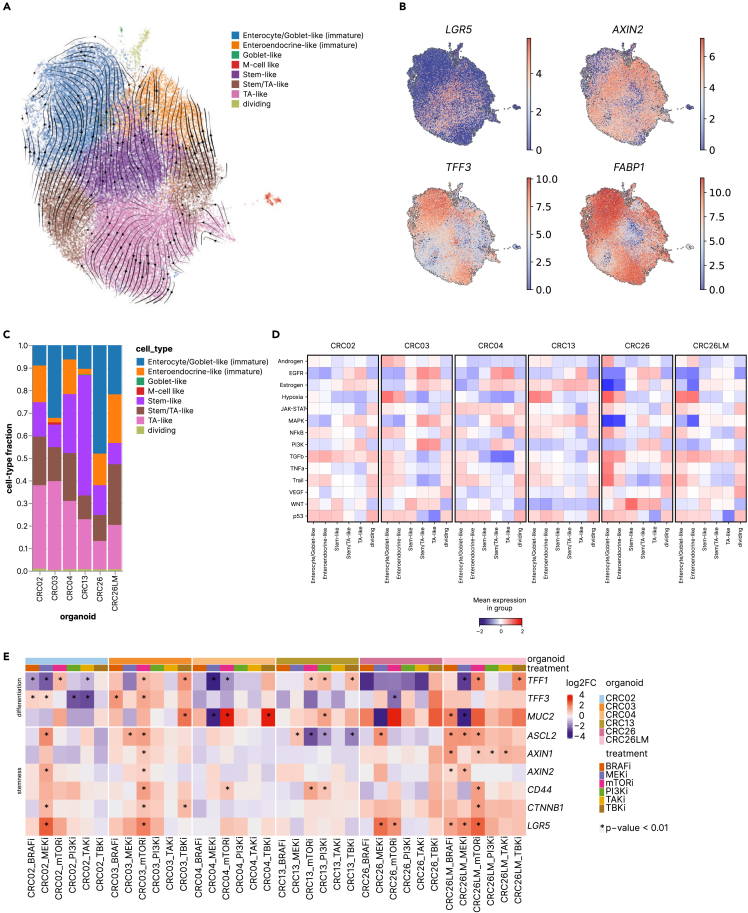
Single-cell analysis of PDOs from CRC patients (A) UMAP plot of batch-corrected scRNA-seq dataset from all organoids, colored by cell type. RNA velocity vectors are projected on top of the UMAP plot. (B) UMAP plot from (A) colored by gene expression (log(CPM)) of the markers for stem cells (*LGR5*), WNT target (*AXIN2*), goblet cells (*TFF3*), and enterocytes (*FABP1*). (C) Cellular composition of the PDOs as measured by scRNA-seq. (D) Analysis of cancer pathways activation in specific epithelial cell types using PROGENy.^38^ (E) qPCR measurements represented in a heatmap of stem cell and differentiation gene markers following treatments with different kinase inhibitors for 72 h. Each drug treatment was performed in triplicates. Fold change in expression of target genes was calculated by 2^−ΔΔCT^ method^39^ using DMSO control for normalization, and *GAPDH* as an endogenous control. ∗p < 0.01; Gene expression fold change values were tested for normality using Shapiro-Wilk test, which showed no deviation from normality. Differences in mean fold change between treated and control were computed by one-way ANOVA with *post hoc* Dunnet’s test. The resulting p values were corrected for false discovery rate (Benjamini-Hochberg) for the number of target genes.

We generated transcriptomic profiles from 37,924 cells (after quality control and filtering) which clustered according to the PDOs with CRC26 (primary tumor) and CRC26LM (liver metastasis) being the closest (Figure S5A). We identified 8 clusters (Figure 5A) which were annotated using curated panels of genes (Figure 5B; Figures S5B and S5C). The PDOs included moderately differentiated stem-like cells, TA-like cells, goblet-like cells, and M-like cells according to the respective markers (Figure 5C; Figures S5B and S5C). The PDOs were highly heterogeneous with respect to the fractions of different cell types, with TA-like cells being most and M-like cells being least abundant (Figure 5C). Analyses of RNA velocity indicated cell hierarchies according to the cancer stem cell hypothesis, with stem cells at the apex, giving rise to TA progenitor cells that undergo differentiation into several cell lineages (Figure 5A). Using a compendium of pathway-responsive gene sets, we then assigned activities of 14 canonical cancer pathways to the individual cell types. The cancer pathway activities were variable between the secretory cells and other cell types, and between the PDOs (Figure 5D). For example, MAPK activity was consistently low in enterocyte/goblet-like and enteroendocrine-like cells, and activated in stem/TA-like and TA-like cells. As CRC26 had a high proportion of enterocyte/goblet-like and enteroendocrine-like cells, this may explain the low and heterogeneous effects of inhibitors on MAP kinases in CRC26 compared to other PDOs (see Figure 3C).

### Kinase inhibitors modulate stemness and differentiation pathways

The phenotypic and differentiation plasticity of the tumor cells shown here can have profound effects on the tumor formation, malignant progression, and response to therapy. For example, it has been shown that MEK inhibitors activate WNT signaling and induce stem cell plasticity in CRC.^37^ Similarly, experimental evidence was provided showing that therapies targeting the MAPK pathway can redirect developmental trajectories of CRC and can be associated with therapy resistance.^40^ However, both studies focused on the inhibitors targeting kinases within the canonical MAPK pathway, i.e., MEK and EGFR/BRAF/MEK. Given the extensive pathway crosstalk in our PDOs, we asked what is the effect of other inhibitors on the phenotypic and differentiation plasticity. We therefore carried out experiments and treated the PDOs for 72 h with the used kinase inhibitors (BRAFi, MEKi, mTORi, PI3Ki, TAKi, TBKi) and analyzed expression of markers for stemness and differentiation with qPCR. The results show heterogeneous effects of the MEKi and BRAFi confirming previous studies.^37^^,^^40^ Moreover, these effects were also observable for other inhibitors including mTORi, PI3Ki, TBKi, and TAKi (Figure 5E). For example, in CRC02 MEKi induced upregulation of the stemness markers *LGR5, CTNNB1, AXIN2*, and *ASCL2* (Figure 5E), whereas in CRC03 mTORi induced both upregulation of all stemness and differentiation markers with the exception of *MUC2* (Figure 5E). Similar diversity was observed for other kinase inhibitors and other PDOs.

### Quantifying heterocellular signaling crosstalk using spatial single-cell proteomics profiling of tumors

**Figure 6:**
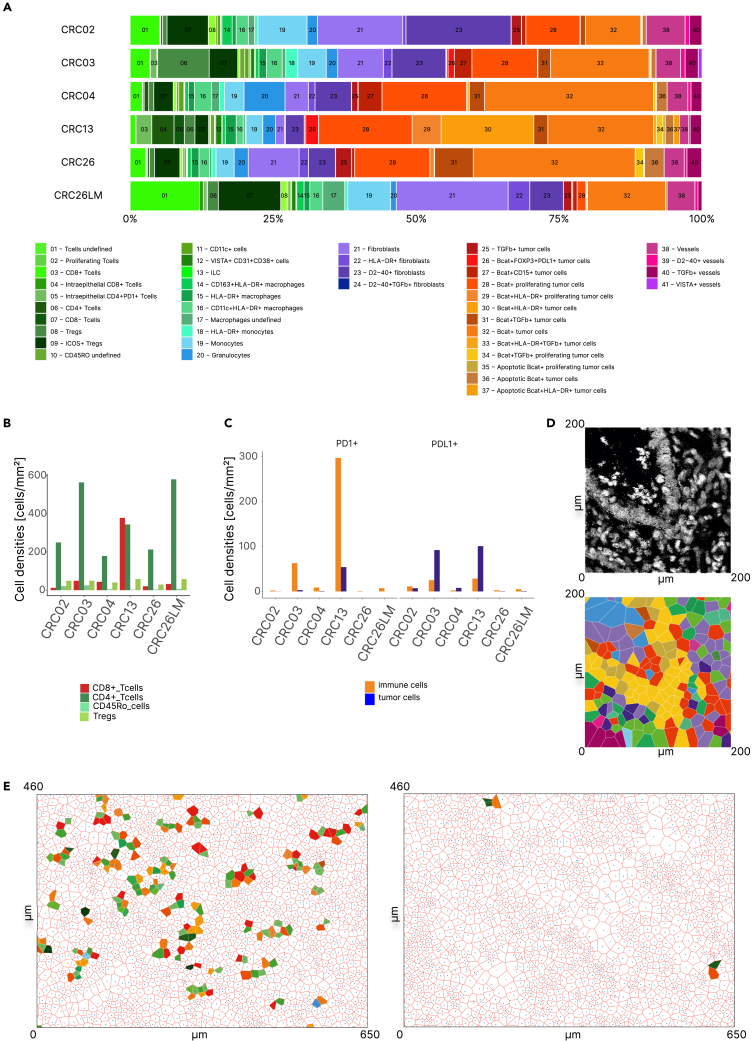
Spatial proteomics of tumor samples using imaging mass cytometry (A) Cellular composition of the TME using 41 cell phenotypes from six tumor tissues from the respective patients. (B) Cell densities of CD8^+^, CD4^+^, CD45RO^+^, and Tregs. (C) Cell densities of PD1^+^ tumor cells and PD-L1^+^ immune cells. (D) Example subsection (200 × 200 μm) of cell neighborhood analysis using Voronoi diagrams. Upper panel: original image. Lower panel: map following cell phenotype identification and building of Voronoi diagrams. (E) Example subsection (650 × 460μm) of the cell neighborhood analysis for PD1^+^ tumor cells and PD-L1^+^ immune cells interactions in CRC13 (left) and CRC03 (right).

Several PDOs showed upregulation of immune pathways following treatment with specific kinase inhibitors, suggesting possible synergistic effects of kinase inhibitors with ICBs. An effective antitumor response following combination therapy of kinase inhibitors and anti-PD-1 or anti-PD-L1 antibodies requires both the presence of CD8^+^ T cells in the tumor microenvironment (TME) and CD8^+^ T cell-tumor cell interactions. We therefore used IMC-based multidimensional imaging of the tumor samples to quantify the densities of immune cell subpopulations and identify heterocellular interactions. We previously developed and evaluated a panel of antibodies for IMC (Table S4) on formalin-fixed, paraffin-embedded (FFPE) samples for a comprehensive overview of the TME and cancer-immune cell interactions,^41^ including lineage and functional immune cell markers, surrogates of cancer cell states (proliferation, apoptosis), and structural markers (epithelium, stroma, vessels) (Figure S6A). We used this panel and FFPE samples from five primary tumors and one liver metastasis of the CRC patients. Following data-driven identification of single-cell phenotypes (Figure S6B), segmentation, and image analysis, we identified 197,454 cells and quantified the densities of five major classes: myeloid, lymphoid, epithelial, fibroblasts, and endothelial cells, which could be further granulated into 41 different cell types (Figure 6A). The cell densities were highly heterogeneous, with CD8^+^ T cells being most abundant in MSI CRC (Figure 6B; Figure S6C). The analyses of the co-expression of immunomodulatory molecules PD-1, PD-L1, ICOS, LAG3, TIM3, and IDO showed heterogeneous populations of immune and tumor cells (Figures 6C and S6D). The densities of PD-1+ immune cells and PD-L1+ tumor cells were highest in tumors from patients CRC03 and CRC13 (Figure 6C), suggesting that these patients are candidates for immunotherapy with anti-PD-1/anti-PD-L1 antibodies.

In order to generate higher-order information beyond cell densities, we investigated spatial cell-cell interactions. We applied cell neighborhood analysis by defining cell nuclei and associating polygons (Voronoi diagram) to each nucleus (Figure 6D), thereby allowing cells of different sizes and distances to be assessed as neighbors.^42^ We used a permutation approach to identify pairwise interactions between cell phenotypes that occurred more or less frequently than expected by chance. This spatial information revealed a number of significant cell-cell interactions, with cell pairs being close neighbors (cell-cell attraction) (Figure S6E) or distant neighbors (cell-cell avoidance) (Figure S6F). Cellular attractions across all tumors were detectable within the lineages of myeloid, lymphoid, epithelial, fibroblasts, and endothelial cells as well as within the classes (Figure S6E). Importantly, neighborhood analysis of PD-L1+ cells and PD1+ cells (defined as direct neighborhood of at least one PD-L1+ tumor cell with at least one PD1+ immune cell) showed that in patient CRC13 there were PD-L1+ tumor cells/PD-1+ immune cells interactions whereas in patient CRC03, despite the relatively high densities of both PD-L1+ tumor cells and PD-1+ immune cells, there were no significant cell-cell interactions (Figures 6C–6E; Table S5). Hence, based on the spatial interaction analysis only patient CRC13 would be amenable for a therapy with anti-PD1 or anti-PD-L1 antibodies.

## Discussion

We developed a functional precision oncology approach using PDOs and quantitative phosphoproteomic profiling and applied this method to demonstrate the feasibility of dissecting tumor cell signaling in individual CRC patients. The information content that can be extracted from these datasets is superior compared to the information content obtained using alternative approaches. Static (i.e., unperturbed) approaches using biopsies or surgical specimens coupled with phosphoproteomic analysis of tumor tissues^43^^,^^44^ resemble the assessment of the steady state of the phosphoproteome and are of limited value for inferring kinase signaling networks. Previous functional approaches using phosphoproteomic measurements to construct cancer signaling networks employed cell lines^45^^,^^46^ and were based on mathematical models that are inherently limited to a small number of molecular interactions.^45^ Recently developed platform using *ex vivo* tumor fragments^47^ could be a viable alternative to the PDOs; however, given the limited amount of material that can be obtained, the phosphoproteome coverage is substantially reduced and the number of possible drugs that can be tested highly restricted. Hence, the “next-generation” functional tests shown here enable comprehensive investigation of the intrinsic CRC biology for successfully personalizing treatment.

The results of our functional precision profiling provide new biological insights and have important translational relevance. First, and most importantly, we show that the patient-specific rewiring of the kinase signaling network is modestly affected by mutations in CRC. Our results suggest that the responses to targeted therapy are additionally determined by non-genetic mechanisms such as those conveyed by phenotypic plasticity.^48^ Single-cell RNA sequencing of the PDOs showed heterogeneous pathway activation in epithelial cell subsets, further supporting the notion of non-genetic mechanisms determining cellular response to drug treatment. We also provide experimental evidence that kinase inhibitors targeting canonical and non-canonical pathways modulate stemness and differentiation pathways, implicating that also repurposed drugs are re-routing developmental trajectories of CRC. This finding is supported by a growing body of literature suggesting that cancer phenotypes and the responses to therapy are determined by non-genetic mechanisms, in addition to the mutation-driven mechanisms commonly considered. For example, a CRC classification system previously proposed associates epithelial cellular phenotypes like stem-like, Goblet-like, or enterocyte cells with responses to cetuximab and standard-of-care chemotherapy.^49^ Similarly, recent work using PDX models showed that EGFR inhibition in CRC tumors induces Paneth-like phenotypic rewiring,^50^ suggesting that cellular plasticity is shaping drug response in cancer. Hence, *in vivo* data using preclinical models and clinical data from large cohorts provide additional evidence for the importance of CRC tumor cell plasticity for the response to targeted therapy. In fact, phenotypic plasticity and disrupted differentiation have been recently proposed as discrete hallmark capability of cancer.^48^

Second, we show that kinase inhibitors can induce profound off-target effects resulting in the modulation of both oncogenic and immune-related pathways. These off-target effects might explain lack of efficacy of targeted therapies as well as failure of combination therapies with ICBs. Off-target effects due to signaling crosstalk, feedback, and feedforward mechanisms, as well as signaling network adaptations, have been previously reported in a variety of cancers and model systems.^31^ However, predicting such off-target effects of specific kinase inhibitors for individual patients based on static multi-omic measurements is not possible. Hence, information-rich assays based on perturbation experiments and phosphoproteomic measurements as presented here are required.

Third, complementing our functional precision profiling with extrinsic information from histology using IMC of the primary tumors enabled us to quantify spatial heterocellular crosstalk and tumor-immune cell interactions and hence provides rationale for combination therapy with ICBs. Thus, our *tour de force* work based on functional precision profiling and single-cell spatial analysis might serve as a blueprint for developing next-generation functional precision oncology platforms for predicting combination therapy response in individuals with metastatic CRC and possibly also other cancers.

### Limitations of the study

Our work has several limitations that can be addressed in future studies. One limitation of our method for inferring kinase network topologies is the use of literature-mined networks. Literature-mined networks are biased toward well-known kinases and pathways as evident by the number of kinases used in our downstream analyses. Given the limited annotations in the public repositories, we were able to use about 10% of the phosphoproteomic data (approx. 600 phosphopeptides out of 6,000 measured). A promising method for revealing new information on kinase-kinase relationships based on chemical phosphoproteomics was recently published^32^ and can be used to infer additional kinase-kinase interactions. Another limitation of our study is the small number of patients and PDOs. Large-scale efforts are needed to investigate big cohorts of patients and potentially identify patterns for patient stratification. Global analysis of the phosphoproteomic data here showed clustering according to patients rather than treatments. Hence, it is intriguing to speculate that there is a limited number of signaling states that could be consequently exploited to stratify patients and ultimately inform therapy. Noteworthily, we used only bulk phosphoproteomic data and could not assign signaling pathways/states to specific epithelial cell subsets. However, technological developments using ultra-high sensitive mass spectrometers are improving^51^ and could in the near future enable single-cell proteome measurements to gain insights into the cellular heterogeneity. Finally, due to the limitations of the phosphoproteomic measurements and the requirement for large amounts of material, we used only a single time point perturbation. Analysis of multiple time points would be extremely valuable, and we advocate that this type of studies can be carried out using more targeted approaches. Our study may serve as blueprint for in-depth investigation based on multiple time points that would be necessary to establish a diagnostic platform.

In summary, the conceptual advances and the insights from the deep molecular and cellular phenotyping we show here challenge the notion that the information flow following kinase inhibition occurs only within specific signaling cascades. We also provide a unique resource of high-quality multi-omics and multi-modal data as well as the corresponding living biobank that can be exploited for both investigation of intrinsic biology of CRC cells as well as the development of novel methods for interrogating intra- and intercellular crosstalk. Finally, our multi-modal profiling approach could provide the basis for the development of a platform for informing precision (immuno-) oncology in CRC.

## STAR★Methods

### Key resources table

**Table:** 

REAGENT or RESOURCE	SOURCE	IDENTIFIER
**Antibodies**		

Anti-chromogranin A	Santa Cruz Biotechnology	Cat#sc-393941; RRID:AB_2801371
Anti-lysozyme	Abcam	Cat#ab108508; RRID:AB_10861277
Anti-mucin 2	Santa Cruz Biotechnology	Cat#sc-515032; RRID:AB_2815005
Anti-EpCAM	R&D	Cat#AF960-SP; RRID:AB_355745
Anti-Ki67	Abcam	Cat#ab92742; RRID:AB_10562976
Anti-Lgr5	Abcam	Cat#ab75732
Anti-SOX9	Sigma	Cat#AB5535; RRID:AB_2239761
Anti-Phalloidin	Abcam	Cat#ab176759
Goat anti-rabbit Alexa Fluor 488	Thermo Scientific	Cat#A11034; RRID:AB_2576217
Goat anti-rabbit Alexa Fluor 594	Thermo Scientific	Cat#A11037; RRID:AB_2534095
Goat anti-mouse Alexa Fluor 488	Thermo Scientific	Cat#A-11029; RRID:AB_2534088
Donkey anti-goat Alexa Fluor 546	Thermo Scientific	Cat#A-11056; RRID:AB_2534103
Donkey anti-rabbit Alexa Fluor 568	Thermo Scientific	Cat#A10042; RRID:AB_2534017
Anti-GAPDH	Santa Cruz Biotechnology	Cat#sc-47724; RRID:AB_627678
Anti-phospho mTOR	Cell Signaling	Cat#5536T; RRID:AB_10691552
Anti-phospho AKT	Cell Signaling	Cat#4060S; RRID:AB_2315049
Anti-phospho ERK1/2	Cell Signaling	Cat#4377S; RRID:AB_331775
Anti-phospho-p38 MAPK	Invitrogen	Cat#36-8500; RRID:AB_2533281
Anti-phosho-MEK1	Invitrogen	Cat#MA5-32165; RRID:AB_2809454
Anti-mTOR	Cell Signaling	Cat#2983; RRID:AB_2105622
Anti-AKT	Cell Signaling	Cat#4691; RRID:AB_915783
Anti-ERK1/2	Cell Signaling	Cat#4695; RRID:AB_390779
Anti-MEK1	Cell Signaling	Cat#2352; RRID:AB_10693788
Anti-p38	Cell Signaling	Cat#8690; RRID:AB_10999090

**Biological samples**		

Human colorectal cancer tissue	This paper	N/A
Human liver metastasis tissue	This paper	N/A
Human blood samples	This paper	N/A

**Chemicals, peptides, and recombinant proteins**		

Advanced DMEM/F12	Thermo Scientific	Cat#12634028
B27 supplement	Thermo Scientific	Cat#17504044
Glutamax	Thermo Scientific	Cat#3550061
HEPES	Sigma	Cat#H0887
Penicillin-Streptomycin	Sigma	Cat#P4333
Noggin conditioned medium	In-house production	N/A
R-spondin conditioned medium	In-house production	N/A
N-acetyl-L-cysteine	Sigma	Cat#A9165
Recombinant human EGF	Peprotech	Cat#AF-100-15
A83-01	Tocris	Cat#2939
Y-27632 dihydrochloride	Abmole	Cat#M1817
SB202190	Sigma	Cat#S7067
Primocin	Invivogen	Cat#ant-pm-2
DMEM	Thermo Scientific	Cat#21969035
Fetal Bovine Serum	Sigma	Cat#F7524
G 418 disulfate salt	Sigma	Cat#A1720
Zeocin	Thermo Scientific	Cat#R25001
Trypsin-EDTA	Sigma	Cat#T4174
TrypLE Express Enzyme	Thermo Scientific	Cat#12604013
Trypan Blue solution	Sigma	Cat#T8154
Geltrex™ LDEV-Free Reduced Growth Factor Basement Membrane Matrix	Thermo Scientific	Cat#A1413202
Cell Recovery Solution	Corning	Cat#7340107
Recovery Cell Culture Freezing Medium	Thermo Scientific	Cat#12648010
Liberase™ DH Research Grade	Roche	Cat#5401054001
StemPro™ hESC SFM	Thermo Scientific	Cat#A1000701
RBC Lysis Buffer	Biolegend	Cat#B420301
Dimethyl sulfoxide	Sigma	Cat#D8418
DPBS	Thermo Scientific	Cat#14190169
AZD6244	Biomol	Cat#LKT-S1846.1
AZD6482	Biomol	Cat#Cay15250-1
AZD8055	Eubio	Cat#SYN-1166-M001
BX-795 hydrochloride	Sigma	Cat#SML0694
PLX4720	Biomol	Cat#Cay15142-1
5Z-7-Oxozeaenol	Sigma	Cat#O9890
Recombinant Human TNF-α	Peprotech	Cat#AF-300-01
Triton X-100	Sigma	Cat#T9284
Paraformaldehyd	Sigma	Cat#16005
Vectashield Antifade Mounting Medium with DAPI	Vectrolabs	Cat#H-2000-2
Goat serum	Sigma	Cat#G9023
Complete mini EDTA-free Protease I	Sigma	Cat#11836170001
Phosphatase inhibitor cocktail 3	Sigma	Cat#P0044
Phenylmethanesulfonyl fluoride solution	Sigma	Cat#93482
Phosphatase inhibitor cocktail 2	Sigma	Cat#P5726
iRT peptides	Biognosys	Cat#Ki-3002-1
Sequencing Grade Modified Trypsin	Promega	Cat#V5113
Lysyl Endopeptidase, Mass Spectrometry Grade (Lys-C)	Wako	Cat#121-05063
Tris(2-carboxyethyl)phosphine hydrochloride	Sigma	Cat#C4706
Iodoacetamide	Sigma	Cat#I1149
Urea	Sigma	Cat#33247
Ammonium bicarbonate	Sigma	Cat#09830
Ammonium hydroxide solution	Sigma	Cat#338818
TiO2 Titanspheres	GL Sciences	Cat#5020-75000
Glycolic acid	Sigma	Cat#124737
C8 extraction discs	3M Empore	Cat#12145002
IHC Antigen Retrieval Solution - Low pH	Thermo Scientific	Cat#00-4955-58
SuperBlock (PBS) Blocking Buffer	Thermo Scientific	Cat#37515

**Critical commercial assays**		

PureLink Genomic DNA Mini Kit	Thermo Scientific	Cat#K182001
RNeasy Plus Mini Kit	Qiagen	Cat#74134
SuperScriptIII first-strand synthesis system	Invitrogen	Cat#18080051
Platinum™ SYBR™ Green qPCR SuperMix-UDG w/ROX	Invitrogen	Cat#11744500
BCA Protein Assay Kit	Thermo Scientific	Cat#23225
NEST MicroSpin Columns	The Nest Group, Inc.	Cat#SEMSS18V
NEST UltraMicroSpin columns	The Nest Group, Inc.	Cat#SUMSS18V
Sep-pak tC18	Waters AG	Cat#WAT36820

**Deposited data**		

Baseline expression data	This paper	ProteomeXChange: PXD019124
Total cell lysate in cancer vs. metastasis data	This paper	ProteomeXChange: PXD018922
Perturbation expression data	This paper	ProteomeXChange: PXD018913
Spectral library for phosphopeptides	This paper	ProteomeXChange: PXD018862
Spectral library for total cell lysate	This paper	ProteomeXChange: PXD018835
All processed data	This paper	Zenodo: 7015015 (https://doi.org/10.5281/zenodo.7015015)
RNA-seq data of organoids and corresponding tumor tissue	Farin et al., 2023^18^	EGA accession number: EGAS0000100730

**Experimental models: Cell lines**		

Colorectal cancer organoids lines	This study	N/A
Liver metastasis organoids lines	This study	N/A
Cell line for production of Noggin	Hubrecht Institute	N/A
HA-R-Spondin1-Fc 293T Cells for production of R-spondin	Amsbio	Cat#AMS.RSPO1-CELLS

**Oligonucleotides**		

Primers for qPCR	Table S6	N/A

**Software and algorithms**		

R version 4.1.3	The R Project for Statistical Computing	https://www.r-project.org/
Spectronaut Pulsar Professional+ version 14	Biognosys, Schlieren, Switzerland.	https://biognosys.com
mapDIA version 3.1.0	Teo et al., 2015^52^	https://sourceforge.net/projects/mapdia/
PTM-SEA version 1.9.0	Krug et al., 2019^27^	https://github.com/broadinstitute/ssGSEA2.0
MaxQuant version 1.5.2.8	Max Planck Institute of Biochemistry, Germany	https://www.maxquant.org/
Python version 3.8.8		https://www.python.org/
nf-core RNA-seq workflow version 1.4.2	Ewels et al., 2020^53^	https://github.com/nf-core/rnaseq
scanpy version 1.8.1	Wolf et al., 2018^54^	https://github.com/scverse/scanpy
cellranger version 5.0.0	10x Genomics	https://support.10xgenomics.com/single-cell-gene-expression/software/pipelines/latest/what-is-cell-ranger
velocyto.py version 0.17.17	La Manno et al., 2018^55^	https://velocyto.org/velocyto.py/
nextNEOpi	Rieder et al., 2022^21^	https://github.com/icbi-lab/nextNEOpi

**Other**		

Code for data analyses	This paper	https://github.com/icbi-lab/plattner_2023

### Resource availability

#### Lead contact

Further information and requests for resources and reagents should be directed to the lead contact, Zlatko Trajanoski (zlatko.trajanoski@i-med.ac.at).

#### Materials availability

There are restrictions to the availability of PDOs generated during this study. An MTA is required for the transfer of the material between the Medical University of Innsbruck and another party.

#### Data and code availability

• Data: The processed data supporting the findings of this study (including exome sequencing, RNA-sequencing, proteomics, phosphoproteomics, single-cell RNA-sequencing, high-dimensional TIFF images, single-cell spatial information and phenotypes) are available online at Zenodo (https://doi.org/10.5281/zenodo.7015015). The MS data which were used to generate the SWATH spectral library, the SWATH raw files and the quantitative results from the SWATH-MS analysis reported in this paper have been deposited in the PRIDE proteomics data repository (https://www.ebi.ac.uk/pride/archive/) under the following accession numbers: PXD019124 (baseline expression experiment), PXD018922 (total cell lysate in cancer vs. metastasis), PXD018913 (perturbation expression experiment), PXD018862 (spectral library for phosphopeptides), PXD018835 (spectral library for total cell lysate) and is publicly available as of the date of publication.
• Code: The code used to produce the results of this study is available at https://github.com/icbi-lab/plattner_2023.
• Any additional information required to reanalyze the data reported in this paper is available from the lead contact upon request.

### Experimental model and study participant details

#### Human subjects

Histologically verified primary and metastatic colorectal tumor tissues and blood samples were obtained from patients undergoing surgical resection at the Medical University Hospital of Innsbruck. Samples were obtained from adult female or male patients who were treatment-naïve, with the exception of patient CRC26 who received FOLFOX and cetuximab before surgery. Written informed consent for research was obtained from patients prior to tissue acquisition. The medical ethical committee of the Medical University of Innsbruck approved protocol AN2016-0194 366/4.9 for the establishment of colorectal cancer PDOs cultures. Details regarding the patient’s clinical information are provided in Table S1.

### Method details

#### Tumor cells isolation

Tumors were washed three times with DPBS (Thermo Scientific, Cat#14190169) containing 100 μg/mL Primocin (Invivogen, Cat#ant-pm-2) and 10 mL/L Penicillin-Streptomycin (Sigma, Cat#P4333), minced finely and incubated with 25 ng/mL Liberase (Roche, Cat#5401054001) in StemPro hESC SFM (Thermo Scientific, Cat#A1000701) for 1 h at 37°C. After incubation, StemPro hESC SFM containing 10% FBS (Sigma, Cat#F7524) was added and the mixture was put over a 400 μM and a 100 μM cell strainers (pluriSelect, # 43-50100-51 and 43-50400-03) to remove large fragments. Cells were spun at 1000 rpm for 4 min, pellet was resuspended in 1x RBC Lysis Buffer (Biolegend, Cat#B420301) and incubated for 10 min at room temperature. Cells were spun at 1500 rpm for 5 min and pellet was washed three times with DPBS followed by centrifugation at 1500 rpm for 3 min.

#### PDOs culture

Isolated tumor cells were seeded at a density of 1.5x10^5^ in 30 μL droplets of 70% Geltrex (Thermo Scientific, Cat#A1413202). The composition of PDO culture medium was: Advanced DMEM/F12 (Thermo Scientific, Cat#12634028) supplemented with 10 mM HEPES solution (Sigma, Cat#H0887), 10 mL/L Penicillin-Streptomycin solution, 2 mM GlutaMAX (Thermo Scientific, Cat#3550061), 20% Rspondin conditioned medium, 10% Noggin conditioned medium, 20 mL/L B-27 supplement (Thermo Scientific, Cat#17504044), 1.25 mM N-Acetylcysteine (Sigma, Cat#A9165), 0.5 nM A83-01 (Tocris, Cat#2939), 10 μM SB202190 (Sigma, Cat#S7067), 50 ng/mL human EGF (Peprotech, Cat#AF-100-15), 100 μg/mL Primocin (Invivogen, Cat#ant-pm-2), and 10 μM Y27632 (AbMole, Cat#M1817). PDO culture medium was refreshed every two days. To passage the PDOs, Geltrex was broken with a cell scraper and PDOs were collected in a tube. The PDOs were centrifuged at 1500 rpm for 5 min, medium was removed, the pellet was resuspended in TripLE Express (Thermo Scientific, Cat#12604013) and incubated for 5 min at 37°C. Advanced DMEM/F12 was added and cells were spun down at 1500 rpm for 5 min. The pellet was resuspended in 70% Geltrex and plated in 30 μL droplets on 6 wells-plates (Sarstedt, #83.3920), 4 drops each well. After allowing Geltrex to solidify, PDO culture medium was added to the plates and PDOs were incubated at 37°C with 5% CO2.

#### Perturbation experiments with PDOs

PDOs were cultured and expanded to forty-eight 30 μL culture-droplets for each condition. At 2 h before collection, PDOs were treated with single inhibitors (or DMSO as solvent control) or at 1 h before collection were stimulated with ligand (or solvent control H2O). The following inhibitors and ligand were used as previously evaluated^45^: MEKi AZD6244 (4 μmol/L, Biomol, #LKT-S1846.1), PI3Ki AZD6482 (10 μmol/L, Biomol, #Cay15250-1), mTORi AZD8055 (2 μmol/L, Eubio, #SYN-1166-M001), TBK1i BX-795 (10 μmol/L, Sigma, #SML0694), BRAFi PLX4720 (5 μmol/L, Biomol, #Cay15142-1), TAK1i 5Z-7-Oxozeaenol (5 μmol/L, Sigma, O9890), and TNFα (10 ng/mL, Peprotech, AF-300-01). After treatment, cultivation dishes were placed on ice and PDOs culture-droplets were washed twice with ice-cold DPBS. Culture-droplets were disrupted in Cell Recovery Solution (Corning, #7340107), collected in a tube and incubated for 1 h on ice. After incubation, PDOs were spun at 1500 rpm for 10 min at 4°C, washed twice with ice-cold DPBS and PDO pellets were snap-frozen in liquid nitrogen and stored at −80°C. Perturbation experiments samples were prepared in duplicates.

#### PDOs preparation for proteomics analysis

PDOs were cultured and expanded to six 30 μL culture-droplets for each sample. At the time of collection, cultivation dishes were placed on ice and PDOs culture-droplets were washed twice with ice-cold DPBS. Culture-droplets were disrupted in Cell Recovery Solution (Corning, #7340107), collected in a tube and incubated for 1 h on ice. After incubation, PDOs were spun at 1500 rpm for 10 min at 4°C, washed twice with ice-cold DPBS and PDO pellets were snap-frozen in liquid nitrogen and stored at −80°C. Proteomics experiments samples were prepared in triplicates.

#### DNA and RNA sequencing

PDOs were harvested, snap-frozen and their DNA were extracted using the PureLink Genomic DNA Mini Kit (Thermo Scientific, #K182001) following manufacturer instructions. Germline DNA were extracted from frozen peripheral blood mononuclear cells (PBMCs) using PureLink Genomic DNA Mini Kit (Thermo Scientific, #K182001) following manufacturer instructions. Exome-sequencing was performed using SureSelect all human V6 capture kit and Illumina sequencing (GATC, Konstanz, Germany and GENEWIZ, Leipzig, Germany). Total RNA was isolated from frozen PDO pellets using the RNeasy Plus Mini Kit (Qiagen, #74134) following manufacturer’s instruction and submitted to total transcriptome full-length mRNA sequencing (GATC, Konstanz, Germany, Medical University of Innsbruck).

#### Single cell sequencing

PDO culture-droplets were washed once with warm DPBS, disrupted with a cell scraper and collected in a tube followed by centrifugation at 1500 rpm for 5 min at room temperature. PDO pellets were resuspended in Trypsin-EDTA (Sigma, Cat#T4174) using 500 μL for each culture-droplet used, resuspended 5 times with a 1000 μL tip and incubated for 5 min at 37°C. An equal volume of Advanced DMEM/F12 was added and PDOs were further dissociated mechanically by resuspending 10 times using a 200 μL pipette tip placed on top of a 1000 μL tip. Cells were filtered through a 40 μm cell strainer and centrifuged at 1500rpm for 5 min at 4°C. Supernatant was removed and cell pellets were resuspended in 1 mL ice-cold 0.04% BSA in DPBS. Cells were counted with a hemocytometer (Marienfeld Neubauer, Cat#0640010) and viability was assessed using Trypan-blue solution (Sigma, Cat#T8154). Single cell suspensions of freshly isolated cells were converted to indexed scRNAseq libraries, using the Chromium Single Cell 3’GEX V3.1 technology from 10x Genomics, aiming for 8.000 cells per library. The resulting Libraries were sequenced with Illumina Novaseq technology (sequencing performed at Genewiz, Leipzig, Germany).

#### Immunofluorescence

PDO samples for immunofluorescence were prepared as described.^56^ Briefly, PDOs were freed from Geltrex by incubation in Cell Recovery Solution (Corning, #7340107) for 1 h on ice, fixed in 4% PFA (Sigma, #16005) in PBS for 1 h at RT and permeabilized in 1% Triton X-100 (Sigma, #T9284) for 30 min at RT. PDOs were incubated in Blocking Buffer (10% goat serum (Sigma, #G9023), 0.2% Triton X-100, 5% BSA in PBS) for 1 h at RT and with primary antibodies in Blocking Buffer overnight at 4°C. PDOs were washed twice with PBS, incubated with secondary antibodies in Blocking Buffer for 2 h at RT in the dark, washed twice with PBS and mounted in Vectashield Antifade Mounting Medium with DAPI (#H-2000-2). Following primary antibodies were used: anti-chromogranin A (Santa Cruz Biotechnology, #sc-393941, 1:500), anti-lysozyme (Abcam, #ab108508, 1:500), anti-mucin 2 (Santa Cruz Biotechnology, #sc-515032, 1:500), anti-EpCAM (R&D, #AF960-SP, 1:20), anti-Ki67 (Abcam, #ab92742, 1:500), anti-Lgr5 (Abcam, #ab75732, 1:100), anti-SOX9 (Sigma, #AB55351:500), Phalloidin-iFluor 647 (Abcam, #ab176759, 1:1000). All secondary antibodies were used 1:800. Immunofluorescence images were captured with an Operetta CLS High-Content Analysis System (PerkinElmer).

#### Western blotting

PDOs were lysed in RIPA buffer with 1x EDTA-free protease inhibitor cocktail (Sigma, #11836170001), 1% v/v phosphatase inhibitor cocktail (Sigma, #P0044) and 2mM PMSF (Sigma, #93482). Protein concentration was measured by BCA Protein Assay Kit (Thermo Scientific, #23225), proteins were separated on 7%, 10% or 12% precast polyacrylamide gels (NuPAGE) following the manual. Blotting was performed using the Invitrogen Novex XCell SureLock Blot-Modul (Invitrogen) according to the manufacturer instructions. Following primary antibodies were used according to manufacturer’s protocol: GAPDH (Santa Cruz Biotechnology, #sc-47724, 1:1000 and Invitrogen, #AM4300, 1:10000), phospho-mTOR (Cell Signaling, #5536T, 1:2000), phospho-AKT (Cell Signaling, #4060S, 1:1000), phospho-ERK1/2 (Cell Signaling, #4377S, 1:1000), phosho-MEK1 (Invitrogen, #MA-32165, 1:1000), phospho-p38 MAPK (Invitrogen, #36-8500, 1:1000), mTOR (Cell Signaling, #2983, 1:1000), AKT (Cell Signaling, #4691, 1:1000), ERK1/2 (Cell Signaling, #4695, 1:1000), MEK1 (Cell Signaling, #2352, 1:1000), p38 MAPK (Cell Signaling, #8690, 1:1000). Chemiluminescence was recorded using the Image Quant Las4000 imaging system (GE Healthcare).

#### mRNA expression analysis of selected genes by RT-qPCR

PDOs were cultured in 6 well flat bottom plates and treated with kinase inhibitors (5 μM BRAFi, 4 μM MEKi, 2 μM mTORi, 10 μM PI3Ki, 5 μM TAKi, 0.25 μM TBKi) and vehicle control (DMSO) for 72 h. After the treatment, organoid pellets were harvested by washing twice in cold PBS, snap frozen in liquid N2 and stored at −80°C before RNA extraction. Total RNA was isolated using RNeasy Plus Mini Kit (Qiagen, cat#: 74134). cDNA was synthesized using SuperScriptIII first-strand synthesis system for RT-PCR (Invitrogen) with 1 μg of total RNA as a template. Quantitative PCR (qPCR) was performed in MicroAmp Optical 384-Well Reaction Plates with Barcode (Applied Biosystems, cat#: 4309849) on ViiA 7 Real-Time PCR System (Applied Biosystems) using Platinum SYBR Green qPCR SuperMix-UDG w/ROX (Invitrogen, cat#: 11744500). The final volume for qPCR reaction was 6 μL containing 4 ng of cDNA, 0.2 μM of each primer and 1X Platinum SYBR Green qPCR SuperMix. Cycling conditions for qPCR were as follows: 2 min at 50°C, 10 min at 95°C followed by 40 cycles of 15 s at 95°C and 60 s at 60°C. Primers used for qPCR are listed in Table S6.

#### Sample preparation for mass spectrometric analyses

Pelleted and frozen PDOs were lysed in 8 M Urea (Sigma, #33247) in 100 mM ammonium bicarbonate (Sigma, #09830) and with sonication for 10 min. For the perturbation experiments, 1:100 phosphatase inhibitor cocktails (Thermo Scientific #P5726 and Sigma #P0044) were added to the lysis buffer. To reduce and alkylate the disulfide bonds, the lysate was reduced using 5 mM tris(2-carboxyethyl)phosphine (TCEP) for 30 min at 37°C and alkylated using 40 mM Iodacetamide (IAA) for 45 min at 25°C in the dark. The protein amount was measured using the Bicinchoninic acid (BCA) assay (Thermo Scientific, #23225) and the appropriate protein amount (60 μg for baseline expression experiments and 1 mg for perturbation experiments) was digested with LysC (1:100, Wako, #121-05063) for 4 h and Trypsin (1:75, Promega, #V5113) overnight. For the digestion with LysC and Trypsin, the samples were diluted to 6 M and 1.5 M Urea (Sigma, #33247) in 100 mM ammonium bicarbonate respectively using 100 mM ammonium bicarbonate (Sigma, #09830). The digestion was stopped the following day by acidification with trifluoroacetic acid to pH 2–3.

For the baseline expression experiments, the digested peptides were desalted using NEST C18 MicroSpin columns by washing with 2% acetonitrile 0.1% trifluoroacetic acid and eluting with 50% acetonitrile 0.1% trifluoroacetic acid. The eluted peptides were dried in a vacuum concentrator, reconstituted in 60 μL 2% acetonitrile 0.1% formic acid in H2O, and spiked with iRT peptides (Biognosys, #Ki-3002-1) prior to injection into the mass spectrometer.

For the perturbation experiments, the digested peptides were desalted using Waters Sep-pak C18 columns by washing with 0.1% trifluoroacetic acid in H2O and eluting with 50% acetonitrile and 0.1% trifluoroacetic acid in H2O. The eluted peptides were subsequently dried in a vacuum concentrator. Before drying fully, an aliquot (1:20) was taken for the corresponding total cell lysate samples. The aliquot was dried and the peptides were dissolved in 2% acetonitrile and 0.1% formic acid in H2O and spiked with iRT peptides (Biognosys, #Ki-3002-1) prior to injection into a mass spectrometer. The remaining part of the sample was destined for phosphoenrichment and dried in a vacuum concentrator.

To enrich for phosphopeptides, the peptides were first dissolved in a tube with loading buffer (1 M glycolic acid, 5% trifluoroacetic acid, 80% acetonitrile in H2O) by shaking 10 min and sonicating for 10 min. For phosphoenrichment, stage tips were constructed placing two C8 plugs into an empty 300 μL tip. These stage tips were placed in a tube using a connector and centrifuged on a table top centrifuge at around 800 g for all subsequent washes. The stage tips were washed with 200 μL methanol to condition the filter. To the washed tips, 80 μL TiO2 bead slurry (2.5 mg TiO2 beads in 50% acetonitrile, 0.1% trifluoroacetic acid) were added and the beads were equilibrated with 200 μL loading buffer. The peptides were loaded by starting with a low centrifugation force of 100 g that was progressively increased until all peptides were loaded. The loaded peptides were washed once with 100 μL loading buffer, once with 100 μL 80% acetonitrile and 0.1% trifluoroacetic acid in H2O, and once with 100ul 50% acetonitrile and 0.1% trifluoroacetic acid in H2O. The peptides were eluted with 250 μL 0.3 M NH3OH and a subsequent elution of 20 μL 50% acetonitrile and 0.1% trifluoroacetic acid in H2O to elute peptides from the filter paper. The phosphopeptides were eluted directly in a tube with trifluoroacetic acid to reach pH 2. The phosphopeptides were then desalted using NEST UltraMicroSpinTM C18 columns and eluted with 50% acetonitrile, 0.1% trifluoroacetic acid in H2O. The buffer was evaporated in a vacuum concentrator and the peptides were dissolved in 2% acetonitrile and 0.1% formic acid in H2O and spiked with iRT peptides (Biognosys, #Ki-3002-1) prior to injection of samples into a mass spectrometer.

#### Acquisition of samples using mass spectrometry

For the perturbation experiments, the peptides were analyzed on an Orbitrap Fusion Lumos mass spectrometer (Thermo Scientific, San Jose, CA) connected to an Easy-nLC 1200 (Thermo Scientific, San Jose, CA) HPLC system. Between 1 μL and 4 μL of peptide solution was separated by nano-flow liquid chromatography using a 120 min gradient from 5 to 37% buffer B in buffer A (Buffer A: 2% acetonitrile, 98% H2O, 0.1% formic acid; Buffer B: 80% acetonitrile, 20% H2O, 0.1% formic acid) on an Acclaim PepMap RSLC 75 μm × 25cm column packed with C18 particles (2 μm, 100 Å) (Thermo Scientific, San Jose, CA). The peptides were ionized using a stainless steel nano-bore emitter (#ES542; Thermo Scientific) using 2000 V in positive ion mode.

To build the spectral libraries, the samples were acquired in data-dependent acquisition (DDA) mode. The DDA method consisted of a precursor scan followed by product ion scans using a 3 s cycle time. The precursor scan was an Orbitrap full MS scan (120’000 resolution, 2 × 105 AGC target, 100 ms maximum injection, 350–1500 m/z, profile mode). The product ion scans were performed using Quadrupole isolation and HCD activation using 27% HCD Collision Energy. The Orbitrap was used at 30’000 resolution and setting the RF Lens at 40%. The AGC Target was set to 5 × 105 and 50 ms maximum injection time. Charge states of 2–5 were targeted and the dynamic exclusion duration was 30s.

To quantify the peptide abundance, the samples were acquired in data-independent acquisition (DIA) mode. The DIA method consisted of a precursor scan followed by product ion scans using 40 windows between 400 m/z and 1000 m/z. The precursor scan was an Orbitrap full MS scan (120,000 resolution, 2 × 105 AGC target, 100 ms maximum injection, 350–1500 m/z, profile mode). The product ion scans were performed using Quadrupole isolation and HCD activation using 27% HCD Collision Energy. The Orbitrap was used at 30,000 resolution using a scan range between 200 and 1800 and setting the RF Lens at 40%. The AGC Target was set to 5 × 105 and 50 ms maximum injection time.

For the baseline expression experiments, the peptides were measured on a Sciex TripleTOF 5600 mass spectrometer with a 90 min gradient and the mass spectrometer was operated in SWATH mode. The precursor peptide ions were accumulated for 250 ms in 64 overlapping variable windows within an m/z range from 400 to 1200. Fragmentation of the precursor peptides was achieved by Collision Induced Dissociation (CID) with rolling collision energy for peptides with charge 2+ adding a spread of 15eV. The MS2 spectra were acquired in high-sensitivity mode with an accumulation time of 50 ms per isolation window resulting in a cycle time of 3.5 s. The samples from the different PDOs were injected consecutively in a block design to prevent any possible confounding effects due to deviation in machine performance. CRC03 and CRC26 were acquired at a later time point, but some original samples were reinjected in parallel to assess that the performance of the machine was similar.

#### Building the spectral library for the perturbation experiments

The raw spectra were analyzed using MaxQuant version 1.5.2.8 that matched each spectrum against a FASTA file containing 20,386 reviewed human (downloaded on August 13, 2018 from www.uniprot.org) and iRT peptides and enzyme sequences. Carbamidomethyl was defined as a fixed modification, and Oxidation (M) as variable modifications. Standard MaxQuant settings for Orbitrap were used (e.g., peptide tolerance 20 ppm for first search and 4.5 for main search). In total, two searches were performed involving 54 injections of peptides and they resulted in the identification of 42’424, peptides from 4’239 protein groups, respectively. The four searches were imported into Spectronaut Pulsar (version 14.0.200309.43655 (Copernicus) Biognosys, Schlieren) to build spectral libraries with the following settings: PSM FDR Cut off of 0.01, fragment m/z between 200 and 1’800, peptides with at least 3 amino acids, fragment ions with a relative intensity of at least 5, precursors with at least 5 fragments. Moreover, an iRT calibration was performed with a minimum root-mean-square error of 0.8 and segmented regression. The spectral library for the total cell lysates contained coordinates for 54’551 precursor peptides from 4’223 protein groups. The spectral library for the phosphopeptide contained coordinates for 30’969 precursor peptides from 4’605 protein groups.

#### Extraction of quantitative data from the mass spectrometry spectra

For the perturbation experiments, quantitative data were extracted from the acquired SWATH-MS maps using Spectronaut Pulsar (version 14.0.200309.43655 (Copernicus) Biognosys, Schlieren) (version 14). As SWATH Spectral library, we used our in-house compiled spectral libraries for the PDOs (see above). We used standard settings (they include a dynamic MS1 and MS2 mass tolerance strategy, a dynamic XIC RT Extraction Window with a non-linear iRT calibration strategy, and identification was performed using a precursor and protein Q value cutoff of 0.01). The quantified intensities for each fragment were extracted from 104 (phospho-enriched samples), 99 (total cell lysate) SWATH-MS injections and the fragment intensities were exported for further statistical analysis to R. Only quantities for fragments that have been detected at least two times in a given condition were selected. Further filtering was performed with mapDIA where a standard deviation factor of 2 and a minimal correlation of 0.25 were used to filter for reliable fragments.

For the baseline expression experiments, the SWATH-MS data was quantified using the OpenSWATH workflow on the in-house iPortal platform using the PanHuman Library.^57^ An m/z fragment ion extraction window of 0.05 Th, an extraction window of 600 s, and a set of 10 different scores were used as described before. To match features between runs, detected features were aligned using a spline regression with a target assay FDR of 0.01. The aligned peaks were allowed to be within 3 standard deviations or 60 s after retention time alignment. The data was then further processed using the R/Bioconductor package SWATH2stats.

#### Variant calling, copy number variation and neoantigen prediction

Somatic mutations, copy number alterations, Class I and II HLA types and neoantigens were called by running our previously published neoantigen prediction pipeline nextNEOpi^21^ (version 1.1).

Briefly, we used the pipelines’ default options but enabled automatic read trimming to remove adapter sequence contamination from raw WES and RNAseq reads and we disabled NetChop and NetMHCstab. Further, we created a panel of normals from the healthy PBMCs and used it to identify recurrent technical artifacts in order to improve the results of the variant calling analysis. Finally, predicted neoantigens were filtered and prioritized using the “relaxed” filter set from nextNEOpi.

The MSI status was determined with MSIsensor^58^ and the scores were plotted as bar plots.

#### RNA-sequencing data analysis

Sequence reads were preprocessed and mapped to the human genome GRCh38/hg38 and GENCODE v33 annotations using the nf-core RNAseq pipeline version 1.4.2 (git revision ff4759e).^53^ In brief, reads were mapped using STAR v2.7.1a^59^ and gene expression quantified with RSEM v1.3.3.^60^ Pathway activity scores were estimated from normalized raw counts using the PROGENy (Pathway RespOnsive GENes) method.^38^ CMS subtypes were predicted with the CMScaller R package v.2.0.1 using raw counts as an input. The intrinsic consensus molecular subtypes (iCMS) were predicted as described in the original paper.^23^ In brief, z-scores of log2-transformed TPM values were used as an input for the nearest template prediction function, implemented in the CMScaller package. The template was created from the 715 iCMS marker genes previously defined by Joanito et al.^23^ GO enrichment analysis was performed using the R package ClusterProfiler.^61^ Eight major clusters were defined and annotated by the GO enrichment results. Heatmaps for visualization of RNAseq results were generated using the ComplexHeatmap.^62^

#### Single-cell RNA-sequencing data analysis

The single-cell RNA sequencing reads were mapped to GRCh38-2020-A reference provided by 10x Genomics using the CellRanger pipeline (v5.0.0). Cellranger’s pre-filtered count matrices were loaded into scanpy^54^ and filtered based on the following quality control cutoffs: ≥2000 genes, 2000 ≤ counts ≤75,000, <25% mitochondrial reads. Doublets were removed using SOLO^63^ v0.6.0. The 5000 most highly variable genes (HGVs) were detected with *scanpy.highly_variable_genes* with *flavor=”seurat_v3”* and *batch_key=”organoid”.* Batch effects were removed using scvi-tools v0.11.0^64^ based on the HVGs and using PDOs as the batch key. A neighborhood graph and UMAP embedding^65^ were calculated with scanpy based on the SCVI latent representation and otherwise default parameters. Unsupervised clustering with the leiden-algorithm^66^ based on the SCVI-corrected neighborhood graph (resolution = 0.5) yielded 12 clusters. Marker genes for each cluster were detected using scvi-tools differential gene expression module^67^ and clusters manually assigned to 7 epithelial cell types based on these marker genes. For visualization, gene expression was CPM-normalized and log1p-transformed, before computing an additional (uncorrected) neighborhood graph and UMAP embedding. RNA velocity was estimated using velocyto.py (v0.17.17)^55^ based on cellranger outputs using the *run10x* command and subsequently loaded into scvelo^68^ for visualization. We performed single-cell pathway analysis using PROGENy.^38^ Scores were computed using the *progeny-py* package v1.0.6. The top 1,000 target genes of the progeny model were used, as recommended for single-cell data.

#### Proteomic data analysis

After quality control steps the median intensity value of baseline proteomic triplicates was retrieved for downstream analysis. Non-uniquely identified proteins were also discarded from further analyses. Differential abundance analysis was done using mapDIA version 3.1.0.^52^ Single-sample enrichment analysis of baseline protein expression levels was performed with the GSVA R package version 1.42.0^69^ using HALLMARK gene sets (version 7.5.1) imported with msigdbr 7.5.1 from MSigDB.^70^ All samples, including replicates were transformed to log2 scale and resulting enrichment scores were then averaged per organoid. GO enrichment analysis was performed the same way as for the RNAseq data (8 major clusters). Heatmaps for visualization of proteomics results were generated using the ComplexHeatmap 2.9.0 R package.^62^

#### Correlation between mRNA and protein abundance

In total, the dataset comprises 3723 overlapping genes and proteins, but only those which were present in at least four out of the 12 matching samples were considered for correlation analysis (n = 3536). Log2 transformed TPM-values for mRNA and log2-transformed protein abundances were used to calculate the Pearson correlation coefficient for each gene. The average correlation between mRNA and protein abundance is 0.29. The results were visualized in a histogram using the ggplot2 R package.

#### Protein complexes

The list of manually curated protein complexes was retrieved from Ori et al.^71^ Human protein complexes with a minimum of five proteins were selected and Pearson correlations within complexes were calculated across PDOs. The top 25% of the protein complexes were considered as variable, whereas the bottom 25% were defined as stable protein complexes. For the 26S proteasome the variance was calculated for all members of the complex, ignoring missing values. The results were visualized as a barplot using the ggplot2 R package.

#### Phosphoproteomic data analysis

Phosphopeptide fragment data were prefiltered for intensities above 2000 and peptides with at least five measured fragments. Missing replicate values were replaced by the 20% value of the minimum of the corresponding fragment abundance. Differential abundance analysis was done using mapDIA version 3.1.0.^52^ Protein sequences and gene symbols were retrieved from UniProt^72^ using the UniProt.ws 2.36.5 R package. Phosphopeptide sequences were matched to protein sequences to determine phosphosite positions. If multiple phosphopeptides mapped to the same phosphosite, we selected the site with the higher mean signal in control samples. Phosphosite readouts for kinases directly targeted by the inhibitors we used were taken from the SIGNOR 2.0 database.^28^ Posttranslational modification set enrichment analysis (PTM-SEA) was performed using the ssGSEA 2.0 R script (ssgsea-cli.R) and the PERT-, PATH- and KINASE-signature categories of the PTMsigDB v1.9.0 database^27^ and kinase/phosphatase signatures derived from SIGNOR. We used fold-change-signed log10-transformed FDR values from mapDIA as input scores and protein-centric phosphosite positions as identifiers. Peptides with multiple phosphorylated residues were demultiplexed as suggested in the original publication. Normalized enrichment scores (NES) and global false-discovery-rate-adjusted p values (FDR) were calculated with the number of permutations set to 100000, “area.under.RES” as the test statistic, a weight of one, no additional normalization and a minimum overlap of two measured sites per signature. For the additional analysis of the common treatment effects that are shared across organoids, we used a linear model in limma 3.50.3 with PDO, treatment and their interaction on the log2-transformed normalized phosphosite abundances from mapDIA. The fold-change-signed log10-transformed p values were used as input for PTM-SEA. Heatmaps for visualization of phosphoproteomics results were generated using the ComplexHeatmap 2.9.0 R package.^62^ The numbers of shared differential abundant phosphopeptides were visualized in R with the UpSetR package.

#### Kinase signaling network analysis

Network analysis and visualization was performed with the igraph 1.3.2, tidygraph 1.2.1 and ggraph 2.0.5 R packages.^73^ We mapped normalized enrichment scores of kinase signatures from PTMsigDB to nodes and phosphosite log-fold-changes to edges of the global human SIGNOR 2.0 signaling network (retrieved on 23.04.2021).^28^ Perturbation subnetworks were identified from perturbed nodes (kinase signature FDR ≤ 0.05) and perturbed edges (phosphosite FDR ≤ 0.05). Briefly, we calculated all shortest paths with a maximum length of two edges between perturbed nodes, combined them with perturbed edges and filtered for the largest connected component. The subnetworks were further refined by pruning them of redundant shortest paths according to whether they could be annotated with measured phosphosites. We further combined the resulting treatment-specific subnetworks into PDO-specific networks and calculated degree and eigenvector centralities of all nodes. To check if the mutations occur with the same frequencies as in the global signaling network we tested for overrepresentation of coding mutations using a two-sided Fisher’s Exact Test.

#### Imaging mass cytometry

Four μm tissue sections were placed on silane-coated glass slides, dried overnight at 37°C and stored at 4°C. Carrier-free IgG antibodies (Table S4) were conjugated to purified lanthanide metals with the MaxPar antibody labeling kit and protocol (Fluidigm) as described by Ijsselsteijn et al.^41^ Imaging mass cytometry immunodetection was performed following the protocol described previously by Ijsselsteijn et al.^41^ In short, tissue sections were deparaffinized through immersion in xylene for 3 times 5 min and rehydrated in decreasing concentrations of ethanol. 10x low pH antigen retrieval solution (Thermo Scientific, #00-4955-58) was diluted in purified water and preheated for 10 min in a microwave. The sections were rinsed in unheated 1x low pH antigen retrieval solution, boiled for 10 min in the preheated buffer and cooled down to room temperature for 1 h. Sections were rinsed with PBS-TB (PBS supplemented with 0.05% Tween and 1% BSA) and incubated for 30 min with 200 μL Superblock blocking buffer (Thermo Scientific, #37515). Antibody incubation was split into two steps: a 5 h incubation at room temperature and an overnight at 4°C incubation. The antibody mix for the 5 h incubation was prepared by diluting the first half of antibodies (Table S4) in PBS-TB after which 100 μL of antibody mix was added to the tissue sections and incubated for 5 h at room temperature in a humid chamber. The sections were washed three times for 5 min with PBS-TB and incubated overnight at 4°C in a humid chamber with the remaining antibodies (Table S4) diluted in PBS-TB. The sections were washed three times with PBS-TB and incubated for 5 min at room temperature with 100 μL Intercalator Ir (1.25 μM diluted in PBS-TB). Tissue sections were washed two times for 5 min with PBS-TB and once with purified water for 5 min. Finally, the sections were dried under an air flow and stored at RT until ablation. For each tissue, 8 regions of interest were chosen based on hematoxylin and eosin staining on consecutive sections that were representative for the whole tissue. The Hyperion imaging mass cytometry system (Fluidigm) was calibrated using a 3-element tuning slide (Fluidigm) following the manufacturers settings with an extra threshold of 1500 mean duals detected for 175Lu. In total, 40 ROIs of 1000 × 1000μm were ablated after which 3 ROIs were excluded due to poor quality.

#### Imaging mass cytometry data analysis

Data was exported as .MCD files and for each ROI color TIFF images were created containing the DNA, vimentin and keratin signal using the Fluidigm MCD viewer. These were used to segment the images into nucleus, membrane and background using a random forest classifier in Ilastik.^74^ The exported probability maps were used to create cell masks in cell profiler.^75^ Simultaneously, the MCD files were transformed to .OME.TIFF files using the Fluidigm MCD-viewer. In Ilastik, pixels were assigned to either ‘signal’ or ‘background’ per marker to train a random forest classifier, which was applied to the entire dataset. Data was exported as binary segmentation masks for each marker where the ‘background’ pixels were set to 0 and the ‘signal’ pixels to 1. These signal masks together with the cell masks and ome.tiff files were loaded into ImaCytE^76^ to create FCS files containing per cell the mean pixel intensity for each marker. HSNE clustering on the FCS files of all images was performed in Cytosplore^77^ to generate phenotype clusters which were mapped back onto the images in IMACyte. Each cluster was visually confirmed using the original .MCD files and combined when similar clusters were observed. The phenotype clusters were further processed using R to calculate cell densities (cells/mm2), composition of present immune cells and to investigate the expression of selected markers of interests in specific phenotype clusters for each sample. Threshold of 10% was set for the marker values, i.e., a cell is considered as positive for a certain marker if in at least 10% of its area the marker was positively detected. The Spearman pairwise correlation heatmaps of cell phenotypes were also calculated for each sample separately. To assess the spatial organization and cell-cell interactions Voronoi diagrams were created and all cell-cell interactions (direct neighbors) were counted. To test whether the number of direct interactions of each pairwise cell type combination is significantly different than expected by chance, a Monte Carlo simulation with 1000 iterations was performed in which the location of the cells from the imaged slide was randomly permuted, while keeping the number of cells from each type constant and the overall cellular positions fixed. Then a *Z* score and p value was calculated to assess avoidance or attraction (*Z* score <2, *Z* score >2, p < 0.01). A combined cohort *Z* score was then calculated using the Stouffer’s *Z* score method for overall meta analysis and plotted as heatmaps (Figures S6E and S6F). PD1+/PDL1+ microaggregates (min. 1 PDL1+ tumor cell and min. 1 PD1+ immune cell) were identified by finding connected components in a graph created from the Voronoi diagram. These microaggregates were color marked with the respective cell type colors and plotted as Voronoi diagrams (Figures 6D and 6E). To test if the number of PD1+/PDL1+ microaggregates per tumor was significantly different than expected by chance, we again performed a Monte Carlo simulation as described above and calculated z-scores and p values (Table S5).

### Quantification and statistical analysis

Statistical analysis for qPCR results was performed in GraphPad Prism v9 software and the statistical test used is indicated in the relevant figure legend. For (phospho-)proteomics, single-cell and imaging mass cytometry data analyses the statistical tests used are described in the respective methods details section and have been performed in R or Python.

## Supplemental information

Document S1. Figures S1–S6 and Tables S4‒S6

Table S1. Clinical information and CMS/iCMS predictions, related to Figures 1, 2B, and 2C

Table S2. Coding mutations in inhibitor targets and gene fusions, related to Figure 2A

Table S3. Protein complexes and kinase enrichment scores, related to Figures 2F and 3C
