# Supplementary material for: Functional and spatial proteomics profiling reveals intra- and intercellular signaling crosstalk in colorectal cancer
Source: iScience. 2023 Nov 4;26(12):108399. doi: 10.1016/j.isci.2023.108399 (PMC10692669; doi:10.1016/j.isci.2023.108399)
Supplement: Document S1. Figures S1–S6 and Tables S4‒S6 [file mmc1.pdf]

iScience, Volume *26*

Supplemental information

Functional and spatial proteomics profiling reveals intra- and intercellular signaling crosstalk in colorectal cancer

Christina Plattner, Giorgia Lamberti, Peter Blattmann, Alexander Kirchmair, Dietmar Rieder, Zuzana Loncova, Gregor Sturm, Stefan Scheidl, Marieke Ijsselsteijn, Georgios Fotakis, Asma Noureen, Rebecca Lisandrelli, Nina Böck, Niloofar Nemati, Anne Krogsdam, Sophia Daum, Francesca Finotello, Antonios Somarakis, Alexander Schäfer, Doris Wilflingseder, Miguel Gonzalez Acera, Dietmar Öfner, Lukas A. Huber, Hans Clevers, Christoph Becker, Henner F. Farin, Florian R. Greten, Ruedi Aebersold, Noel F.C.C. de Miranda, and Zlatko Trajanoski

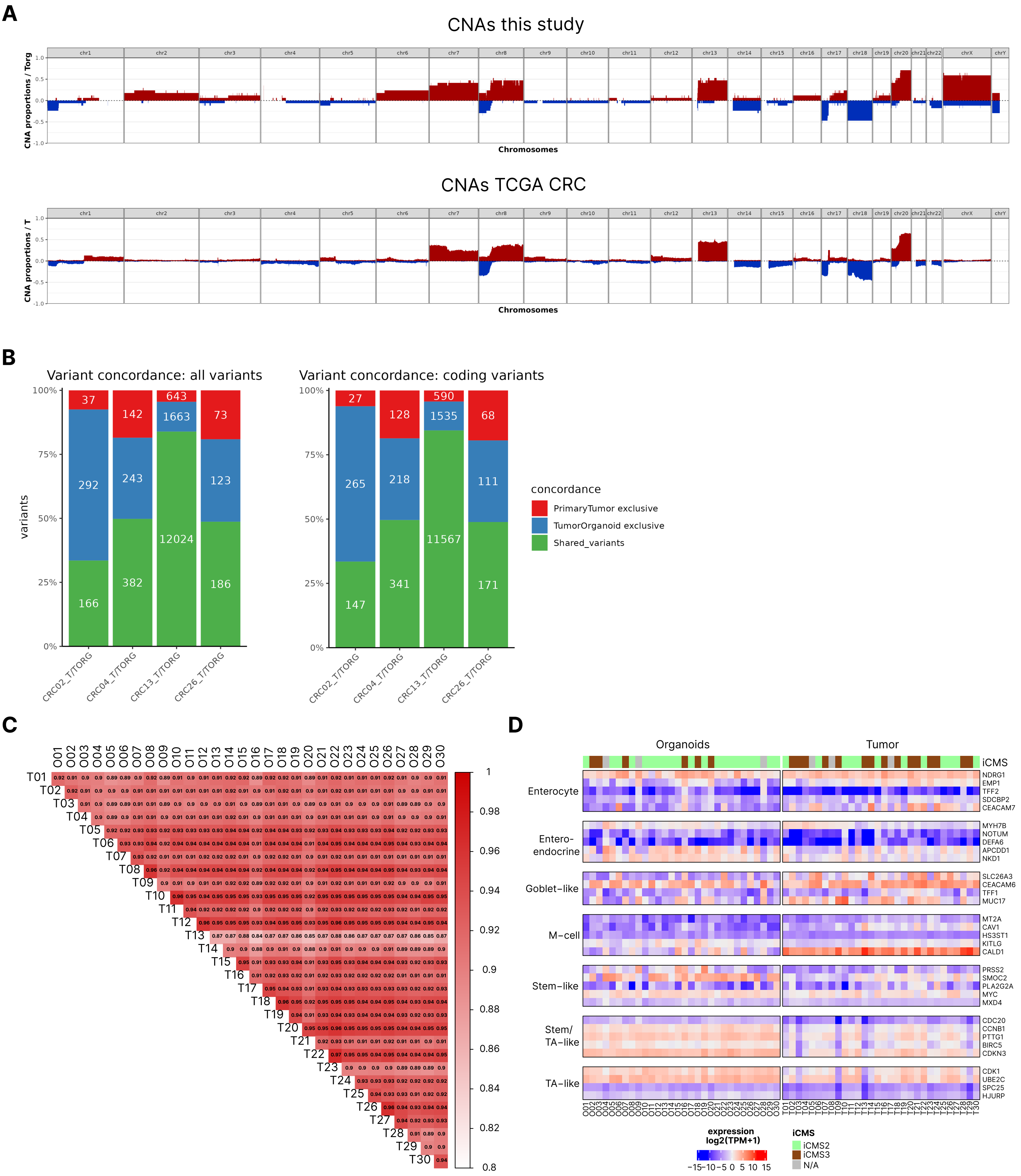

Figure S1. Genomic and transcriptomic characterization of the PDOs (related to Figure 2)
(A) Copy number alteration analysis for PDOs (upper plot) and the TCGA CRC cohort (lower plot). Red: amplifications. Blue: deletions. **(B)** Variant concordance between primary tumor and PDOs showing shared and exclusive variants (left: all variants, right: coding variants only). **(C)** Pearson correlation of RNA expression profiles between organoids and primary tumors from an independent bulk RNA-seq dataset [S1]. **(D)** Comparison of marker gene expression from 30 different organoids and their corresponding tumor tissue. The heat map shows the log2(TPM+1) values for gene signatures used for cell type annotation (see Figure S5).

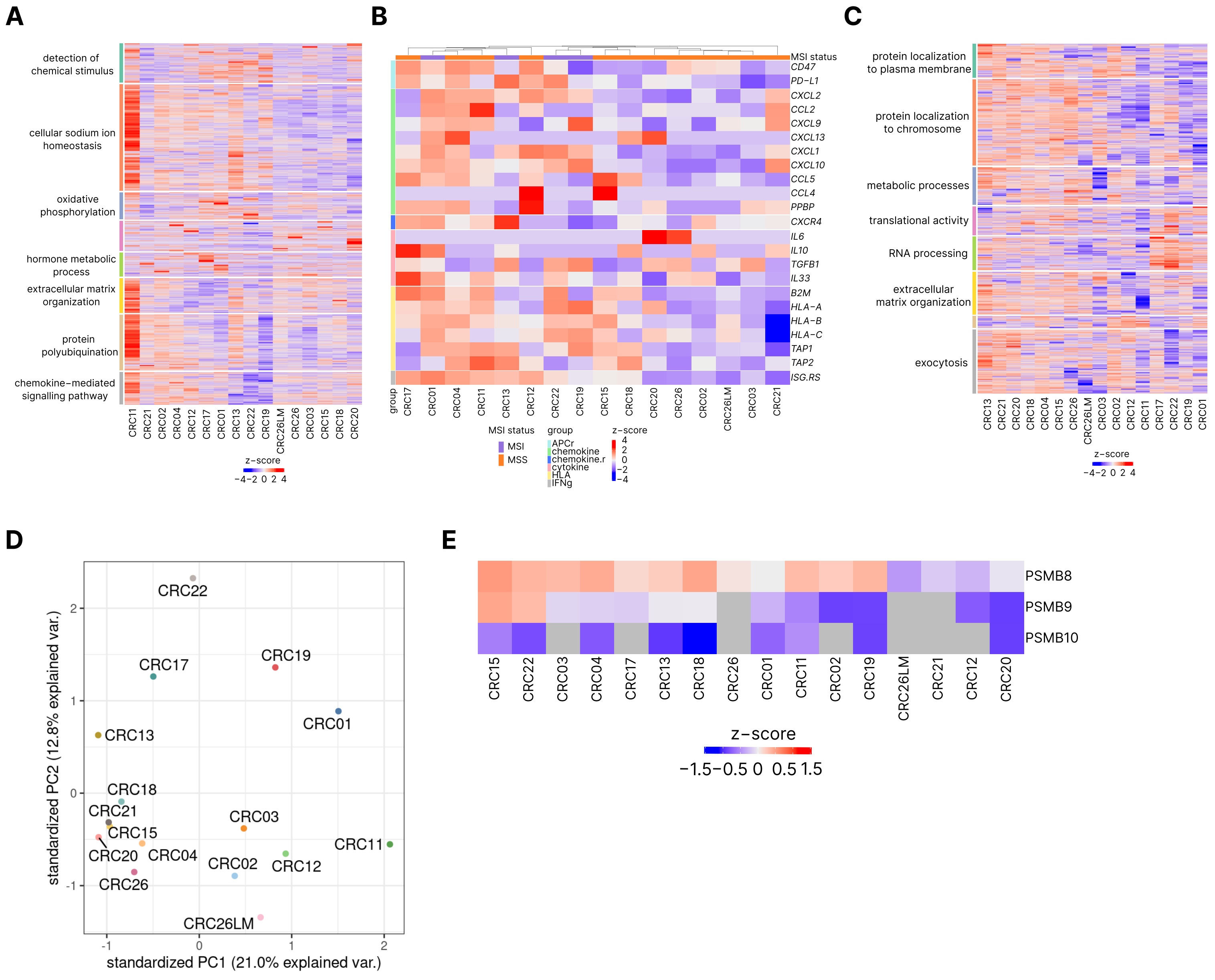

Figure S2. Transcriptomic and proteomic characterization of the PDOs (related to Figure 2)
(A) Heatmap of the steady-state RNA-sequencing data. Eight major clusters were defined and annotated using GO enrichment analysis. The genes are z-scaled and clustered hierarchically, using Pearson correlation as distance and complete linkage. **(B)** Heatmap of immune-related genes grouped as antigen presenting cell receptors (APCr), chemokines, chemokine receptors, cytokines, human leukocyte antigens (HLA) and interferon gamma (IFNg). ISG.RS: interferon metagene for IFNG signature genes [S2]. **(C)** Heatmap of the steady-state proteomics data. Eight major clusters were defined and presented the same way as the gene expressions in (A). **(D)** Principal component analysis of the proteomics data. **(E)** Heatmap representing z-scores of the log2-transformed protein levels of the subunits of the immunoproteasome.

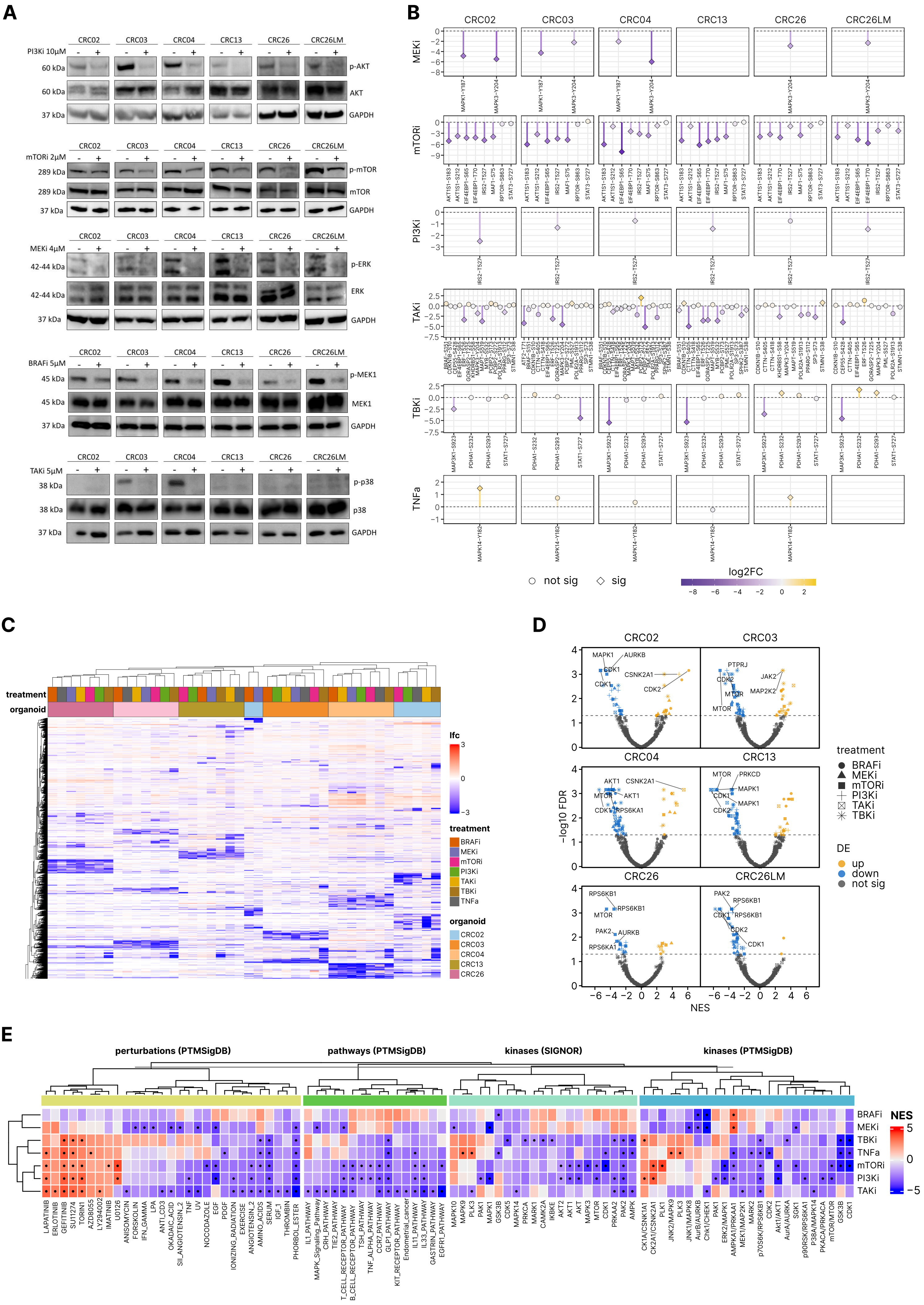

A
CRC02
CRC03
CRC04
CRC13
CRC26
CRC26LM
PI3Ki 10μM
mTORi 2μM
MEKi 4μM
BRAFi 5μM
TAKi 5μM
60 kDa
289 kDa
42-44 kDa
45 kDa
38 kDa
37 kDa
p-AKT
AKT
GAPDH
p-mTOR
mTOR
p-ERK
ERK
p-MEK1
MEK1
p-p38
p38
B
MEKi
mTORi
PI3Ki
TAKi
TBKi
TNFa
not sig
sig
log2FC
C
treatment
organoid
lfc
BRAFi
MEKi
mTORi
PI3Ki
TAKi
TBKi
TNFa
D
-log10 FDR
NES
DE
up
down
not sig
E
perturbations (PTMSigDB)
pathways (PTMSigDB)
kinases (SIGNOR)
kinases (PTMSigDB)
NES

Figure S3. Analysis of the phosphoproteomic data following signaling perturbations (related to Figure 3)
(A) Western blots for selected targets of kinase inhibitors. **(B)** Phosphoproteomics-based log-2-fold-changes in the phosphorylation of selected potential downstream targets of kinase inhibitors or activators following the respective treatments (with FDR<0.05 indicated as significant). **(C)** Heatmap of all log-2-fold-changes in the phosphorylation of residues with FDR<0.05 in at least one treatment, clustered by complete linkage of Euclidean distances. **(D)** Volcano plots of normalized enrichment scores (NES) for kinase activity signatures following treatment of PDOs with specific kinase inhibitors or TNFα. **(E)** Normalized PTM-SEA enrichment scores (NES) of kinase, pathway and perturbation signatures following PDO treatments. Black dots indicate significance (FDR<0.05)

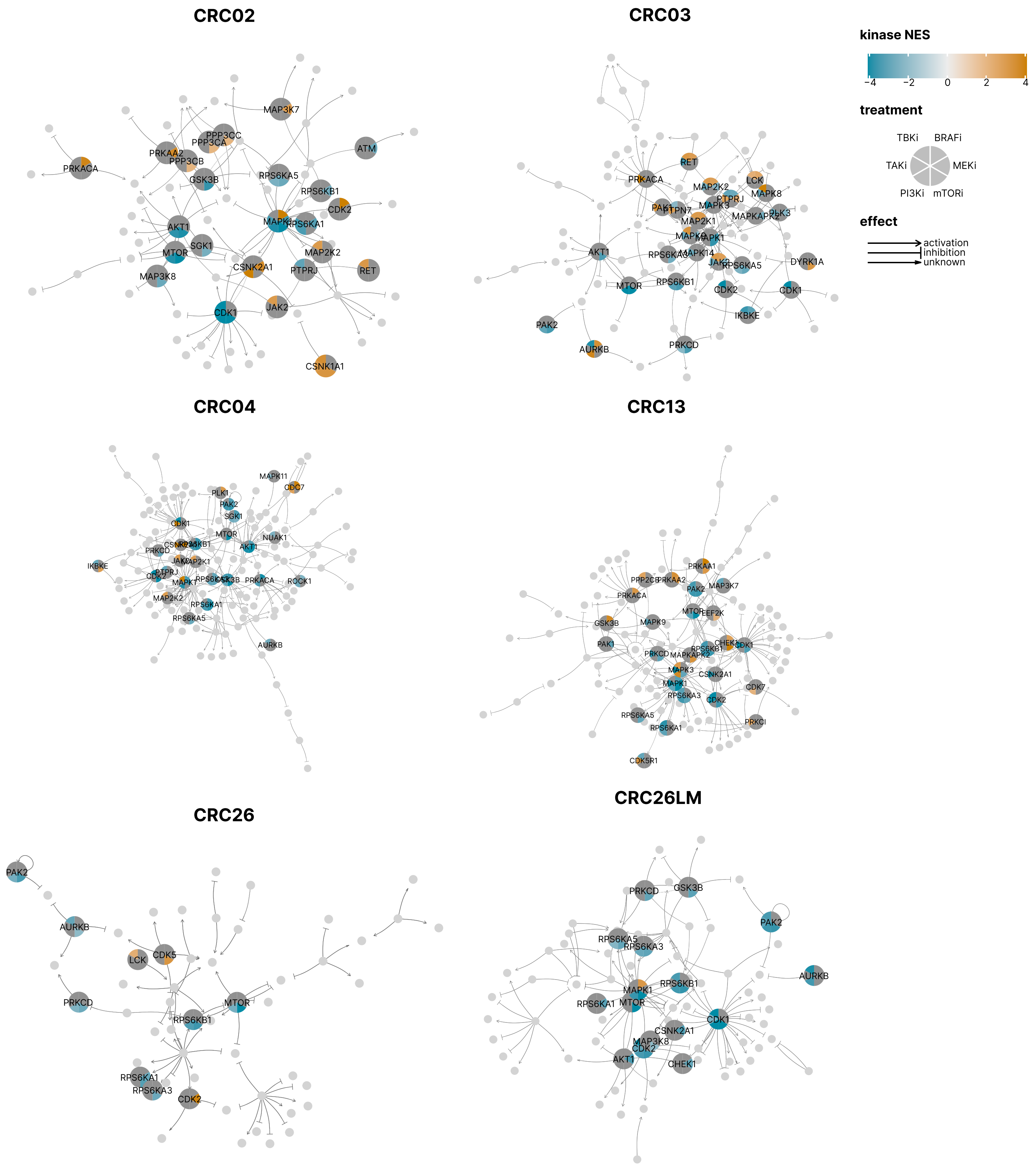

Figure S4. Signaling networks (related to Figure 4A)
Signaling networks of the top inhibitor-perturbed kinases for selected PDOs, extracted from a protein interaction database using the phosphoproteomic perturbation data. Kinase activities (normalized enrichment scores, NES) of significantly (FDR<0.05) perturbed nodes are shown for each treatment. Edge arrows indicate the effects on target nodes.

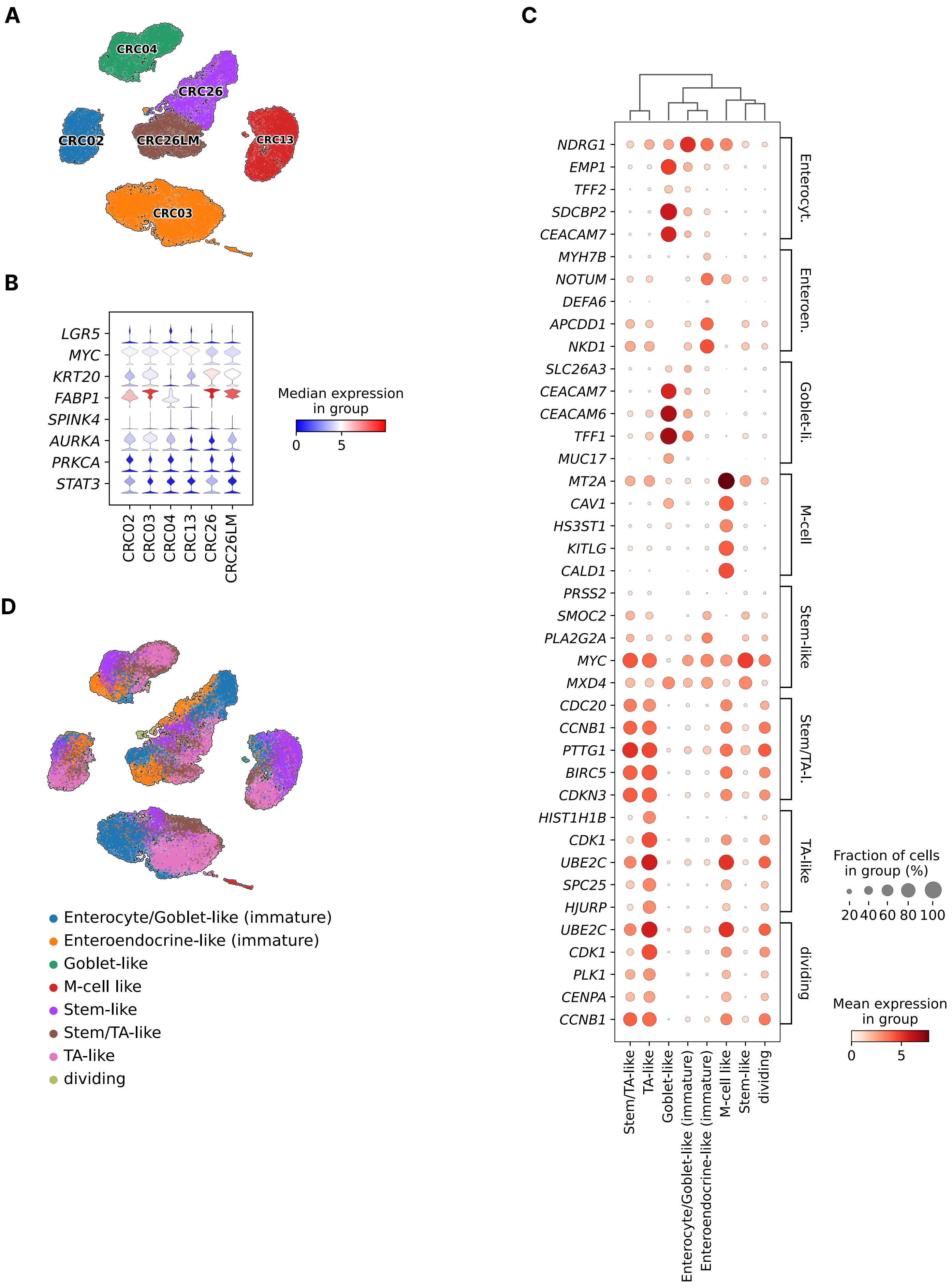

Figure S5. Single-cell RNA-seq analysis of the PDOs (related to Figure 5)
(A) UMAP of the scRNA-seq data, colored by PDO. **(B)** Violin plots for selected markers. **(C)** Dot plots for the markers used for cell annotation. **(D)** UMAP of the RNA-sequencing data, colored by cell type.

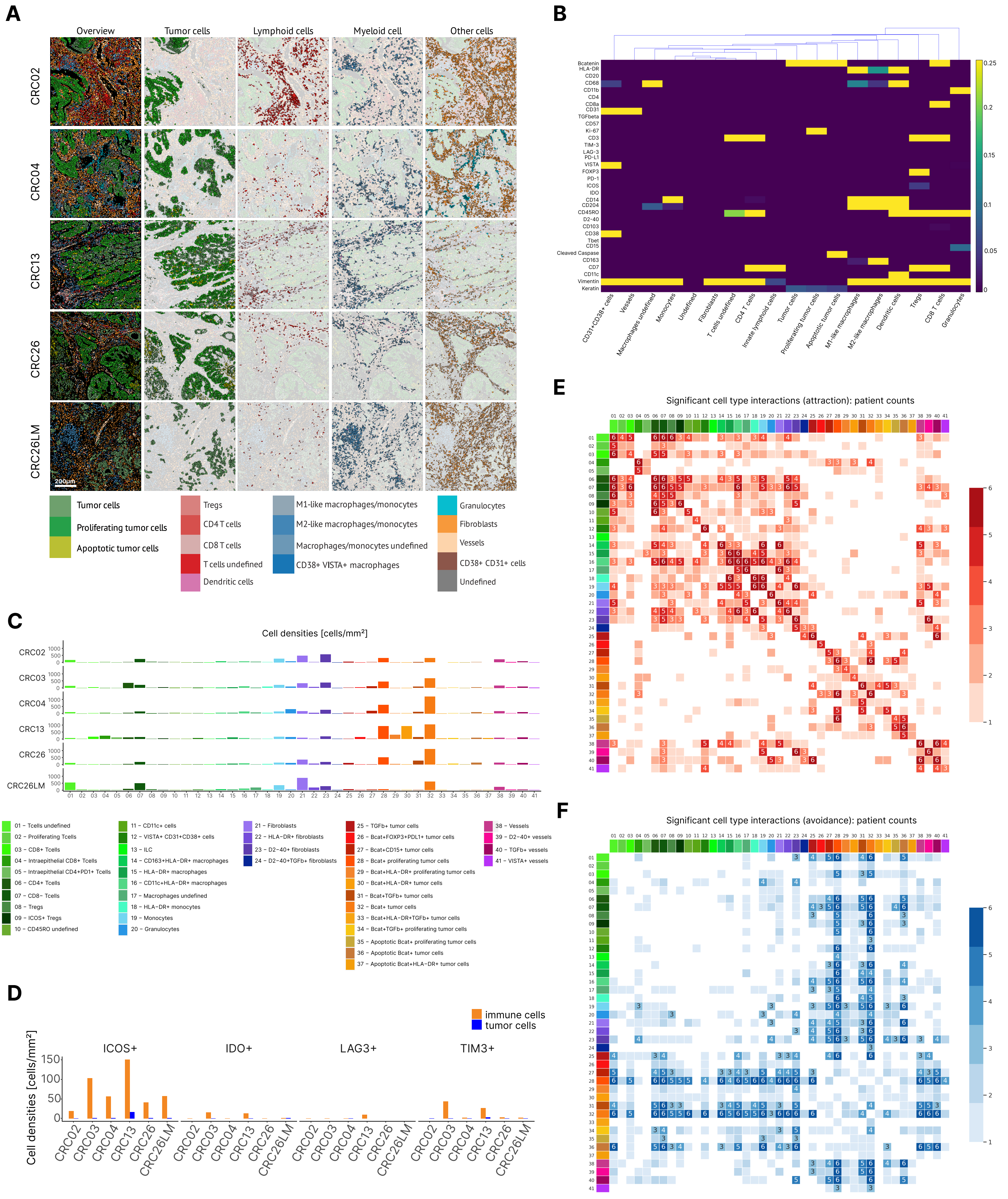

Figure S6. Preprocessing of the imaging mass cytometry data (related to Figure 6)

(A) Example IMC images of the major cell types (pseudo coloured) for selected organoids, showing their heterogeneity. Subgroups of major cell types are coloured in similar colour shades. **(B)** Hierarchical clustering of the measured expression values of 34 selected markers (see also Table S4) used for the phenotyping and recognition of different cell types. **(C)** Cell densities for the four major cell types. **(D)** Cell densities for selected immuno-modulatory molecules. **(E)** Interaction analysis showing cell-cell attraction. Squares are showing the number of PDOs in which significant interactions occur. **(F)** Interaction analysis showing cell-cell avoidance. Squares are showing the number of PDOs in which significant interactions occur.

Table S4. IMC antibody metal combinations (related to Figure 6A)

Target	Clone	Metal	Incubation Time	Temp	Dilution
HLA-DR	TAL 1B5	141Pr	5h	RT	100
CD11b	D6X1N	144Nd	5h	RT	100
CD4	EPR6855	145Nd	5h	RT	50
CD8a	D8A8Y	146Nd	5h	RT	50
TGFbeta	TB21	149Sm	5h	RT	100
TIM3	D5D5R(TM)	154Sm	5h	RT	100
LAG-3	D2G40(tm)	155Gd	5h	RT	50
VISTA	D1L2G(TM)	158Gd	5h	RT	100
PD-1	D4W2J	160Gd	5h	RT	50
ICOS	D1K2T(tm)	161Dy	5h	RT	50
CD14	D7A2T	163Dy	5h	RT	100
CD204	J5HTR3	164Dy	5h	RT	50
CD103	EPR4166(2)	168Er	5h	RT	50
Tbet	4B10	170Er	5h	RT	50
Cleaved-caspase	5A1E	172Yb	5h	RT	100
CD163	EPR14643-36	173Yb	5h	RT	50
CD7	EPR4242	174Yb	5h	RT	100
CD11c	EP1347Y	176Yb	5h	RT	100
B catenin	D10A8	115In	Overnight	4C	100
CD20	H1	142Nd	Overnight	4C	100
CD68	D4B9C	143Nd	Overnight	4C	100
CD31	89C2	147Sm	Overnight	4C	100
CD57	HNK-1 / Leu-7	151Eu	Overnight	4C	100
Ki-67	8D5	152Sm	Overnight	4C	100
CD3	EP449E	153Eu	Overnight	4C	50
PD-L1	E1L3N(R)	156Gd	Overnight	4C	50
FOXP3	D608R	159Tb	Overnight	4C	50
IDO	D5J4E(TM)	162Dy	Overnight	4C	100
CD45RO	UCHL1	165Ho	Overnight	4C	100
D2-40	D2-40	166Er	Overnight	4C	100
CD38	EPR4106	169Tm	Overnight	4C	100
CD15	BRA-4F1	171Yb	Overnight	4C	100
Vimentin	D21H3	194Pt	Overnight	4C	50
Keratin	C11 and AE1/AE3	198Pt	Overnight	4C	50

Table S5. PD1-immune - PDL1-tumor microaggregates (related to Figure 6C-E)

sample_id	# microaggregates	z-score	p-value
CRC02	2	2.52	5.88E-03
CRC03	27	-0.08	4.68E-01
CRC04	10	2.88	2.02E-03
CRC13	780	10.59	1.68E-26
CRC26	0	-0.55	2.93E-01
CRC26LM	6	3.46	2.73E-04

Table S6. qPCR primers (related to Figure 5E)

Target Gene	Forward Primer (5'-3')	Reverse Primer (5'-3')	Ref
ASCL2	GCACCAACACTTGGAGATTTT	AATGGATTCTCTGTGCCCTTAG	[S3]
AXIN1	CTACCTCACATTCCCCGCAC	GATGAGCTCCTCCGCGAACTT	
AXIN2	AGAGCAGCTCAGCAAAAAGG	CCTTCATACATCGGGAGCAC	[S3]
CD44	CAAGCAGGAAGAAGGATGGAT	AACCTGTGTTTGGATTTGCAG	[S3]
CTNNB1	GGTCGAGGACGGTCGGA	CTGTGGTAGTGGCACCAGAAT	
LGR5	TTCCCAGGGAGTGGATTCTAT	ACCAGACTATGCCTTTGGAAAC	[S3]
TFF1	CCCCCGTGAAAGACAGAATTG	GTGTCTAAAATTCACACTCCTCTTC	
TFF3	CTGCTGCTTTGACTCCAGGAT	CAGCTGGAGGTGCCTCAGAA	[S4]
MUC2	AGGATCTGAAGAAGTGTGTCACTG	TAATGGAACAGATGTTGAAGTGCT	[S5]
GAPDH	AGCCACATCGCTCAGACAC	GCCCAATACGACCAAATCC	[S3]

Supplemental References

[S1] Farin HF, Mosa MH, Ndreshkjana B, Grebbin BM, Ritter B, Menche C, Kennel KB, Ziegler PK, Szabó L, Bollrath J, Rieder D, Michels BE, Kress A, Bozlar M, Darvishi T, Stier S, Kur IM, Bankov K, Kesselring R, Fichtner-Feigl S, Brüne B, Goetze TO, Al-Batran SE, Brandts CH, Bechstein WO, Wild PJ, Weigert A, Müller S, Knapp S, Trajanoski Z, Greten FR. Colorectal Cancer Organoid-Stroma Biobank Allows Subtype-Specific Assessment of Individualized Therapy Responses. Cancer Discov. 2023 Oct 5;13(10):2192-2211.
doi: 10.1158/2159-8290.CD-23-0050. PMID: 37489084; PMCID: PMC10551667.

[S2] Benci JL, Xu B, Qiu Y, Wu TJ, Dada H, Twyman-Saint Victor C, Cucolo L, Lee DSM, Pauken KE, Huang AC, Gangadhar TC, Amaravadi RK, Schuchter LM, Feldman MD, Ishwaran H, Vonderheide RH, Maity A, Wherry EJ, Minn AJ. Tumor Interferon Signaling Regulates a Multigenic Resistance Program to Immune Checkpoint Blockade. Cell. 2016 Dec 1;167(6):1540-1554.e12.
doi: 10.1016/j.cell.2016.11.022. PMID: 27912061; PMCID: PMC5385895.

[S3] Zhan T, Ambrosi G, Wandmacher AM, Rauscher B, Betge J, Rindtorff N, Häussler RS, Hinsenkamp I, Bamberg L, Hessling B, Müller-Decker K, Erdmann G, Burgermeister E, Ebert MP, Boutros M. MEK inhibitors activate Wnt signalling and induce stem cell plasticity in colorectal cancer. Nat Commun. 2019 May 16;10(1):2197.
doi: 10.1038/s41467-019-09898-0. PMID: 31097693; PMCID: PMC6522484.

[S4] Yusufu A, Shayimu P, Tuerdi R, Fang C, Wang F, Wang H. TFF3 and TFF1 expression levels are elevated in colorectal cancer and promote the malignant behavior of colon cancer by activating the EMT process. Int J Oncol. 2019 Oct;55(4):789-804.
doi: 10.3892/ijo.2019.4854. Epub 2019 Aug 5. PMID: 31432157; PMCID: PMC6741840.

[S5] VanDussen KL, Marinshaw JM, Shaikh N, Miyoshi H, Moon C, Tarr PI, Ciorba MA, Stappenbeck TS. Development of an enhanced human gastrointestinal epithelial culture system to facilitate patient-based assays. Gut. 2015 Jun;64(6):911-20.
doi: 10.1136/gutjnl-2013-306651. Epub 2014 Jul 9. PMID: 25007816; PMCID: PMC4305344.
